# Estradiol-ERβ2 signaling axis confers growth and migration of CRPC cells through TMPRSS2-ETV5 gene fusion

**DOI:** 10.18632/oncotarget.11355

**Published:** 2016-08-17

**Authors:** Hogyoung Kim, Amrita Datta, Sudha Talwar, Sarmad N. Saleem, Debasis Mondal, Asim B. Abdel-Mageed

**Affiliations:** ^1^ Department of Urology, Tulane University School of Medicine, New Orleans, Louisiana, 70112, USA; ^2^ Department of Pharmacology, Tulane University School of Medicine, New Orleans, Louisiana, 70112, USA; ^3^ Tulane Cancer Center, Tulane University School of Medicine, New Orleans, Louisiana, 70112, USA

**Keywords:** estrogen, ERβ2, TMPRSS2:ETV5, IGF-1R, prostate cancer

## Abstract

Estrogen receptor beta (ERβ) splice variants are implicated in prostate cancer (PC) progression; however their underlying mechanisms remain elusive. We report that non-canonical activation of estradiol (E_2_)-ERβ2 signaling axis primes growth, colony-forming ability and migration of the androgen receptor (AR)-null castration-resistant PC (CRPC) cells under androgen-deprived conditions (ADC). The non-classical E_2_-ERβ2 mediates phosphorylation and activation of Src-IGF-1R complex, which in turn triggers p65-dependent transcriptional upregulation of the androgen-regulated serine protease *TMPRSS2:ETV5a/TMPRSS2:ETV5b* gene fusions under ADC. siRNA silencing of *TMPRSS2* and/or *ETV5* suggests that *TMPRSS2:ETV5* fusions facilitates the E_2_-ERβ induced growth and migration effects via NF-κB-dependent induction of cyclin D1 and MMP2 and MMP9 in PC-3 cells. Collectively, our results unravel the functional significance of oncogenic *TMPRSS2:ETV5* fusions in mediating growth and migration of E_2_-ERβ2 signaling axis in CRPC cells. E_2_-ERβ2 signaling axis may have significant therapeutic and prognostic implications in patients with CRPC.

## INTRODUCTION

Prostate cancer (PC) is the most common form and the second leading cause of cancer-related death among American males [1]. The American Cancer Society estimated 200,800 new cases of PC with an anticipated death rate of 27,540 among American males in 2015 [1]. Despite an initial 12–18 months of regression in response to hormone ablation therapy [2], patients frequently present with relapse of a more aggressive castration-resistant PC (CRPC) [3]. However, the current treatment options are limited because the underlying mechanisms that govern the stepwise transition to CRPC remain elusive.

Estrogen (E_2_) regulates cellular physiology *via* activation of estrogen receptors (ERs) through canonical and non-canonical signaling pathways [4]. In the classical pathway, E_2_ diffuses through the plasma membrane, binds to ERs in the cytoplasm, the ligand-bound ER dimers then translocate to the nucleus and bind estrogen-responsive elements (EREs) on target genes, regulating their signaling. We have reported earlier that E_2_ elicits cell surface activation of ER signaling via a variety of signal transduction pathways [5]. Also, our recent demonstration of a correlation between ERβ expression in prostate tumors and disease progression suggests a potential involvement of ERβ in the development of late-stage PC, especially among African American men [6]. In addition, preclinical studies have shown the use of selective estrogen receptor modulators (SERMs) for the prevention and treatment of CRPC [7] and Nakajima *et al*. have reported that E_2_ antagonists may abrogate ERβ- and KLF5-mediated signaling, and promote cellular proliferation [8]. However, the mechanisms by which ERβ primes PC progression are not fully elucidated.

Prostate cancer is marked with recurrent gene fusions, including *TMPRSS2* and E26 transformation-specific (*ETS*) transcription factors [9]. All *ETS* members share significant sequence homology encompassing 85 amino acids in the C-terminal *ETS* domain and a DNA binding 5′-GGA(A/T)-3′ motif [9]. *G*ene fusion of the 5′-untranslational region of *TMPRSS2* with v-*ETS*, avian erythroblastosis virus E26, oncogene homolog (*ERG*), has been widely reported in PC [9]. In addition to its commonly known fusion with the *ERG* gene, *TMPRSS2* also fuses with other members of the ETS family, such as *ETV1 (also named ER81 for ETS-related 81), ETV4 (ETS variant 4)*, and *ETV5 (ERM for ETS-related molecule)*, in approximately 10% of PCs [10]. The *TMPRSS2:ETS* gene fusions have been shown to play an important role in cellular proliferation [11], migration, and invasion [7]. However, the oncogenic mechanisms of *TMPRSS2:ETV5* gene fusion and their related signaling are still under study.

Evidence from expression profiles in PC cohorts shows an association between *TMPRSS2* gene fusions and estrogen receptor (ER) signaling [12]. However, the mechanisms by which *TMPRSS2* is regulated by estrogen are not delineated. Here we have investigated the molecular mechanisms that underlie the expression of *TMPRSS2* gene and its fusion forms through non-canonical (E_2_-ERβ-Src-IGF-1R) pathway in androgen receptor (AR)-null PC-3 cells.

## RESULTS

### Estradiol stimulates growth of ERβ-expressing AR-null PC-3 cells

**Figure 1:**
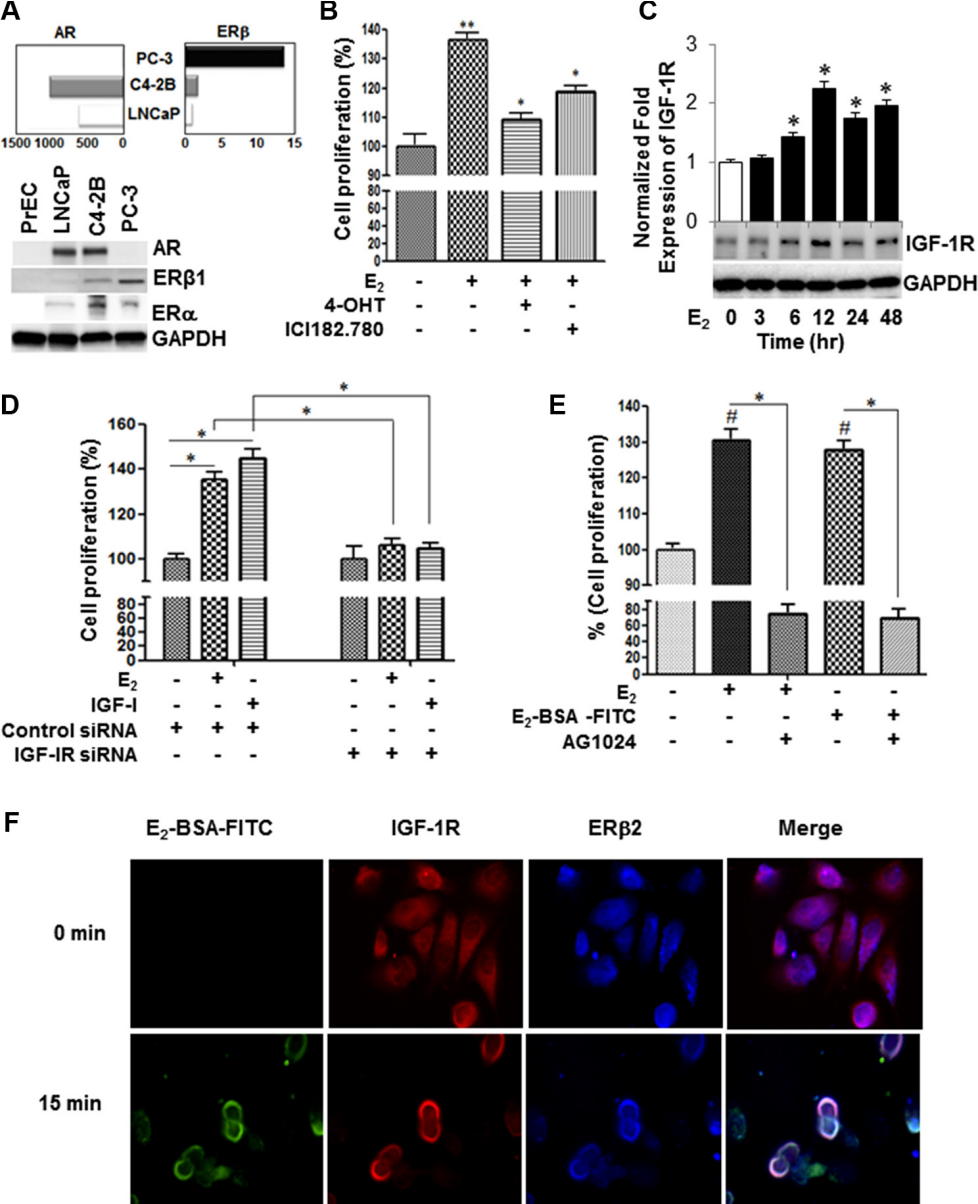
Estrogen induces IGF-1R-dependent cell proliferation through non-canonical activation of ERβ2 in AR-null PC-3 cells (**A**) qRT-PCR and Western blot analyses of AR, ER-β and GAPDH in a panel of PC cell lines. The PC cells were maintained for 48 hours in 10% CS-FBS. Then, the target genes and protein levels were measured by qRT-PCR (upper panel), and Western blot analyses (lower three panels), respectively, in PrEC, LNCaP, C4-2b, or PC-3 cells as described in the Methods section. (**B**) 4-hydroxtamoxifen (4-OHT), a selective estrogen receptor modulator, or ICI182.780 (antiestrogen) inhibited E_2-_ mediated growth stimulatory effects in PC-3 cells. PC-3 cells (5 × 10^3^ cells per well) were incubated with vehicle or 1 uM inhibitor for 48 h. Cell growth was measured by cell counting kit-8 (CCK-8). (**C**) Time-course analysis of IGF-1R protein expression in E_2_-stimulated PC-3 cells. (**D**) PC-3 cells were transiently transfected with *IGF-1R* siRNA or non-targeting control siRNA, after which cells were treated with vehicle, 10 nM E_2_ or 50 ng IGF-I for 48 h and cell proliferation was assessed by CCK-8. (**E**) Selective IGF-1R inhibitor (AG1024; 5 μM) attenuated growth stimulatory effects treated with E_2_ or E_2_-BSA-FITC in PC-3 cells for 48 hours. (**F**) PC-3 cells cultured in chamber slides were stimulated with E_2_-BSA-FITC and then fixed under non-permeabilization conditions, and subjected to immunofluorescence staining with antibodies against ERβ2 and IGF-1R. The E_2_-BSA-FITC (green) stimulation triggered co-localization of IGF-IR (red) and ERβ2 (blue) on the plasma membrane of PC-3 cells. The upper right panel shows that permeabilization is required to detect ERβ in the nucleus. The lower panel shows that under permeabilized conditions, IGF-1R (green) co-localizes with ERβ2 (red) in PC-3 cells. DAPI is indicated in blue. The images were captured using Leica fluorescence microscope. Bar graphs represent mean ± SEM values in triplicates. * and ** denotes significance at *P* < 0.05 and *P* < 0.01, respectively, compared to vehicle-treated cells (*n* = 3).

We examined the constitutive expression of AR and ERβ transcripts and protein levels in normal human primary prostate epithelial cells (PrEC) and a panel of PC cell lines, LNCaP, C4-2B, and PC-3 cells, by qRT-PCR using “pan” PCR primer set. Figure 1A (upper panel) depicts that the constitutive expression of all ERβ transcript levels in the AR-null PC-3 cells are at least 10-fold higher compared to the AR-expressing LNCaP and its isogenic metastatic castration-resistant C4-2B cells. However, C4-2B cells have twice as high endogenous ERβ transcripts compared with its parental LNCaP androgen-dependent cells. The expression of endogenous ERβ and ERα levels in these cell lines were corroborated by immunoblot analysis using pan anti-ERβ antibody (Figure 1A; lower panel). In comparison to PC cells, the protein expression levels of AR and ERβ were very low or undetectable in PrEC cells. The 17β-estradiol (E_2_) stimulated growth of AR-negative PC-3 cells under hormone-deprivation conditions (HDC) (Figure 1B). The selective E_2_ growth stimulatory effect was abrogated by 4-hydroxtamoxifen (4-OHT), a selective estrogen receptor modulator (SERM) or ICI 182,780 (Fulvestrant^®^), an ER antagonist, as measured by cell counting assay kit-8. In contrast, while moderate effect was observed in the CRPC AR-expressing C4-2B, E_2_ triggered no effect on growth in LNCaP cells (*data not shown*).

The role of estrogens in androgen independence has been suggested by the observation that both primary and metastatic PC expresses the ERβ subtypes [13]. Based on our qRT-PCR analysis, constitutive expression of ERβ2 is 3-fold higher in PC-3 than DU-145 and normal prostate epithelial cells (PrEC) (Supplementary Figure S1A). The finding was corroborated by Western blot analysis (Supplementary Figure S1B). Notably, E_2_ did not elicit growth stimulatory effect in DU-145 cells, suggesting that ERβ2, but not ERβ1, is critical to E_2_-induced mitogenic response in PC-3 cells (Supplementary Figure S1C).

### IGF-1R is required for non-canonical E_2_-ERβ2 induced proliferation of PC-3 cells

Because of its potential role in PC progression, we investigated if the expression of IGF-1R is modulated in E_2_-treated PC-3 cells. Our qRT-PCR analysis revealed that E_2_ induces time-dependent transcription of IGF-1R mRNA above the threshold level. The E_2_-induced IGF-1R protein levels reached their maximum levels at 12 h and sustained for at least 48 h after treatment (Figure 1C). A critical outcome of activation of IGF-1R signaling is promotion of cell growth. To this end, we examined if siRNA-mediated silencing of IGF-1R modulates the E_2_ and/or IGF-I induced proliferative responses in PC-3 cells. The siRNA knockdown of IGF-1R resulted in discernible inhibition of E_2_ or IGF-I induced cell proliferation in comparison to vehicle-treated controls or control siRNA transfected cells (Figure 1D). Next, we employed both unconjugated E_2_ and the extracellular non-diffusible fluorescein-labeled E_2_-BSA (E_2_-BSA-FITC) form, capable of preferential binding to ER on plasma membranes, to determine if E_2_ induction of IGF-1R expression and mitogenic response is mediated via genomic or non-canonical pathways. Like E_2_, E_2_-BSA-FITC stimulated growth in PC-3 cells under hormone-deprivation condition (HDC), implicating that ERβ2 may trigger PC-3 cell proliferation via a non-classical pathway (Figure 1E). The growth inhibition by the IGF-1R selective inhibitor (AG1024) in PC-3 cells treated with E_2_ or E_2_-BSA-FITC (Figure 1E) corroborates this notion and further suggests that the non-classical activation of IGF-1R by E_2_ may be pivotal to initiation of mitogenic signaling in these cells. These findings were further validated by confocal immunofluorescence studies to examine if ERβ2 co-localizes with IGF-1R on the plasma membrane within hormone stimulated PC-3 cells. Under non-permeabilized conditions, IGF-1R (red) co-localized with ERβ (blue) on the plasma membrane in PC-3 cells pre-stimulated with E_2_-BSA-FITC (green) compared with untreated cells (0 min) (Figure 1F). Similar results were observed upon replacing secondary antibodies for ERβ2 and IGF-1R or when pan anti-ERβ antibody was employed (Supplementary Figure S2A and S2B, respectively). In contract, ERβ nuclear localization was observed with pan anti-ERβ antibody in E_2_-stimulated PC-3 cells under plasma membrane permeabilization conditions (Supplementary Figure S2C). Collectively, the results suggest that IGF-IR is required for proliferative response mediated by E_2_-ERβ2 axis in PC-3 cells.

### E_2_-ERβ2 signaling axis primes expression and phosphorylation of IGF-1R and NF-κB through activation of Src (p^418^Src) in PC-3 cells

**Figure 2:**
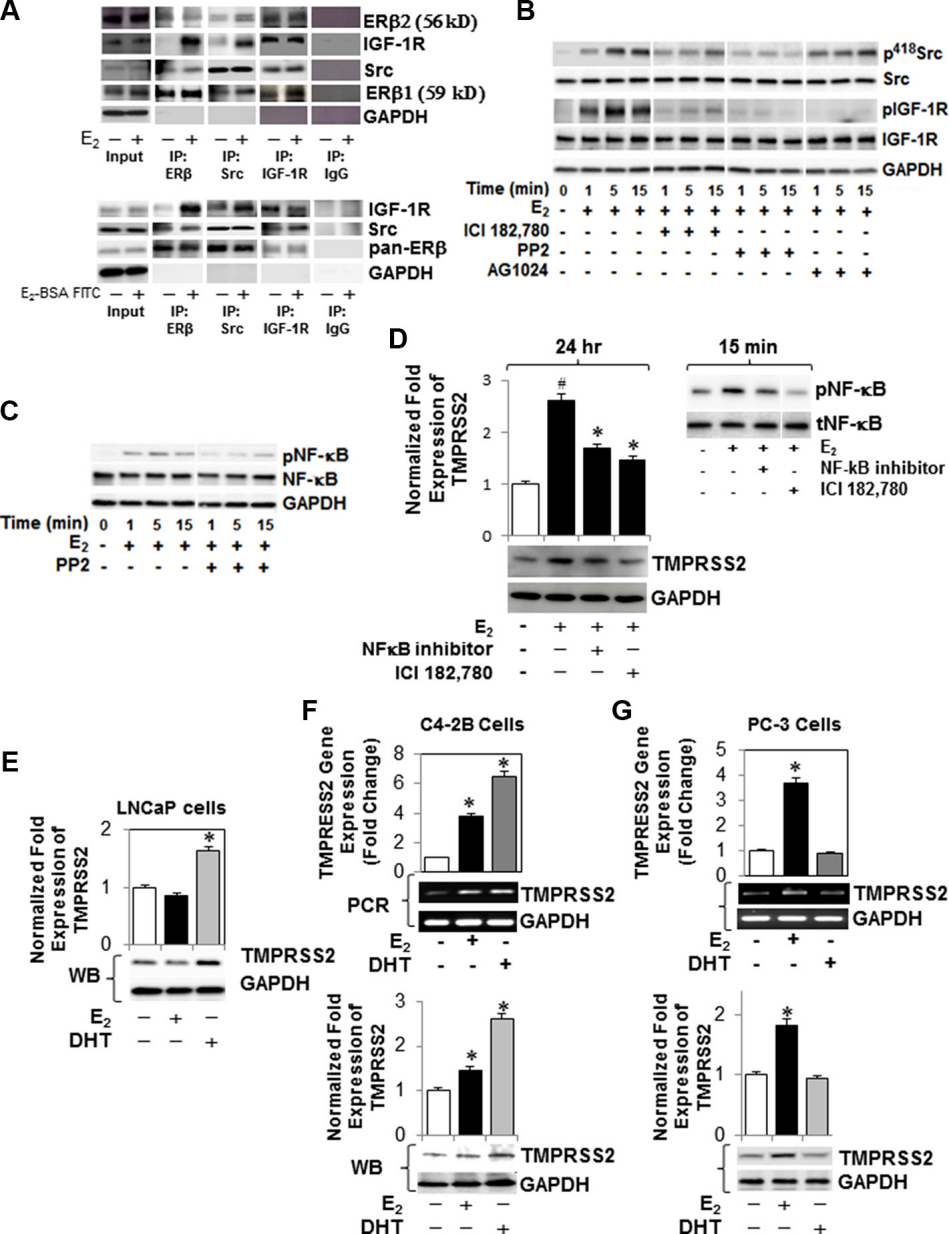
E_2_-ERβ2 signaling axis primes Src (p^418^Src)-dependent activation of IGF-1R and NF-κB and expression of TMPRSS2 in PC-3 cells (**A**) PC-3 cells were treated with vehicle, 10 nM E_2_ (upper panel) or E_2_-BSA-FITC (bottom panel) for 5 min, after which protein extracts were subjected to immunoprecipitation with antibodies against c-Src, IGF-1R, ERβ or IgG control. The immunoprecipitates and 10% input were subjected to immunoblot analysis with antibodies against c-Src, ERβ1 (59 kD), ERβ2 (56 kD), IGF-1R, or GAPDH. For controls, lysates were immunoprecipitated with control IgG followed by immunoblot analysis. (**B**) PC-3 cells treated with E_2_ (10 nM) in the presence of PP2 (selective Src inhibitor), IC1 182,780 or AG1024 were harvested at different time intervals as indicated. Protein lysates were subjected to immunoblot analysis with antibodies against phospho-IGF-1R (p-IGF-1R), IGF-1R, phospho-Src (p^416^ Src), Src, or GAPDH. (**C**) Protein lysates of PC-3 cells treated 10 nM E_2_ at different time intervals, in the presence or absence of PP2, were examined for expression of phospho-NF-κB (pNF-κB), NF-κB, or GAPDH by immunoblot analysis. (**D**) Western blot analysis of TMPRSS2 expression in PC-3 cells treated with E_2_ (10 nM) in the presence or absence of ICI 182,780 (1 μM) or NF-κB activation inhibitor II for 24 hours. Protein extracts with E_2_ (10 nM) for 15 min were prepared and subjected to immunoblot analysis with antibodies against pNF-κB or tNF-κB (inset). (**E**) AR-expressing LNCaP cells were treated with E_2_ (10 nM) or DHT (10 nM) for 24 hours and TMPRSS2 protein levels were measured by immunoblot analysis against GAPDH. (**F**, **G**) C4-2B and PC-3 cells, respectively, were treated with E_2_ (10 nM) or DHT (10 nM) for 12 hours. Cell lysates were analyzed for *TMPRSS2* gene expression by qRT-PCR (upper panel) and conventional RT-PCR (middle panel) and protein level by immunoblot analysis against GAPDH. Bar graphs represent mean ± SEM values in triplicates. * denotes significance at *p* < 0.01 compared to controls (*n* = 3).

Since Src tyrosine kinase (Src) is implicated in the development of cancer metastasis, we sought to examine its recruitment and interaction with ERβ2 and/or IGF-1R on plasma membrane in E_2_-stimulated PC-3 cells. Figure 2A depicts an interaction between c-Src (total), ERβ (pan-antibody) and IGF-1R (total) when measured by co-immunoprecipitation assays in PC-3 cells treated with E_2_ (upper panel) or E_2_-BSA-FITC (lower panels) compared to vehicle-treated cells and control IgG. Next, we assessed whether E_2_-ERβ axis not only confers interaction but also activates IGF-1R and Src in PC-3 cells. As shown in Figure 2B, E_2_ selectively induces phosphorylation of Src (p^418^Scr) in PC-3 cells within 5 min. The activation of Src is nullified by the ER antagonist ICI 182,780, suggesting a direct role for E_2_-ERβ axis in activation of Src. The reversal of Src activation by the selective inhibitor PP2 shows its activation is required for E_2_-induced IGF-1R phosphorylation, which was comparable in effect to the AG1024-mediated inhibition of Src phosphorylation (Figure 2B). In addition, the activation of Src appears to be required for E_2_-mediated activation of NF-κB, as evidenced in PP2-treated PC-3 cells (Figure 2C). Taken together, the results suggest that E_2_ binding to ERβ2 triggers recruitment and activation of c-Src, which in turn interacts with ERβ2 and activates IGF-1R and NF-κB in PC-3 cells under HDC. Supplementary Figure S6A–S6E provide full blots for the Western and immunoprecipitation analyses.

### Activation of E_2_-ERβ2 signaling triggers NF-κB-dependent expression of *TMPRSS2, ETVs* and *TMPRSS2:ETV5* gene fusions in PC-3 cells

**Figure 3:**
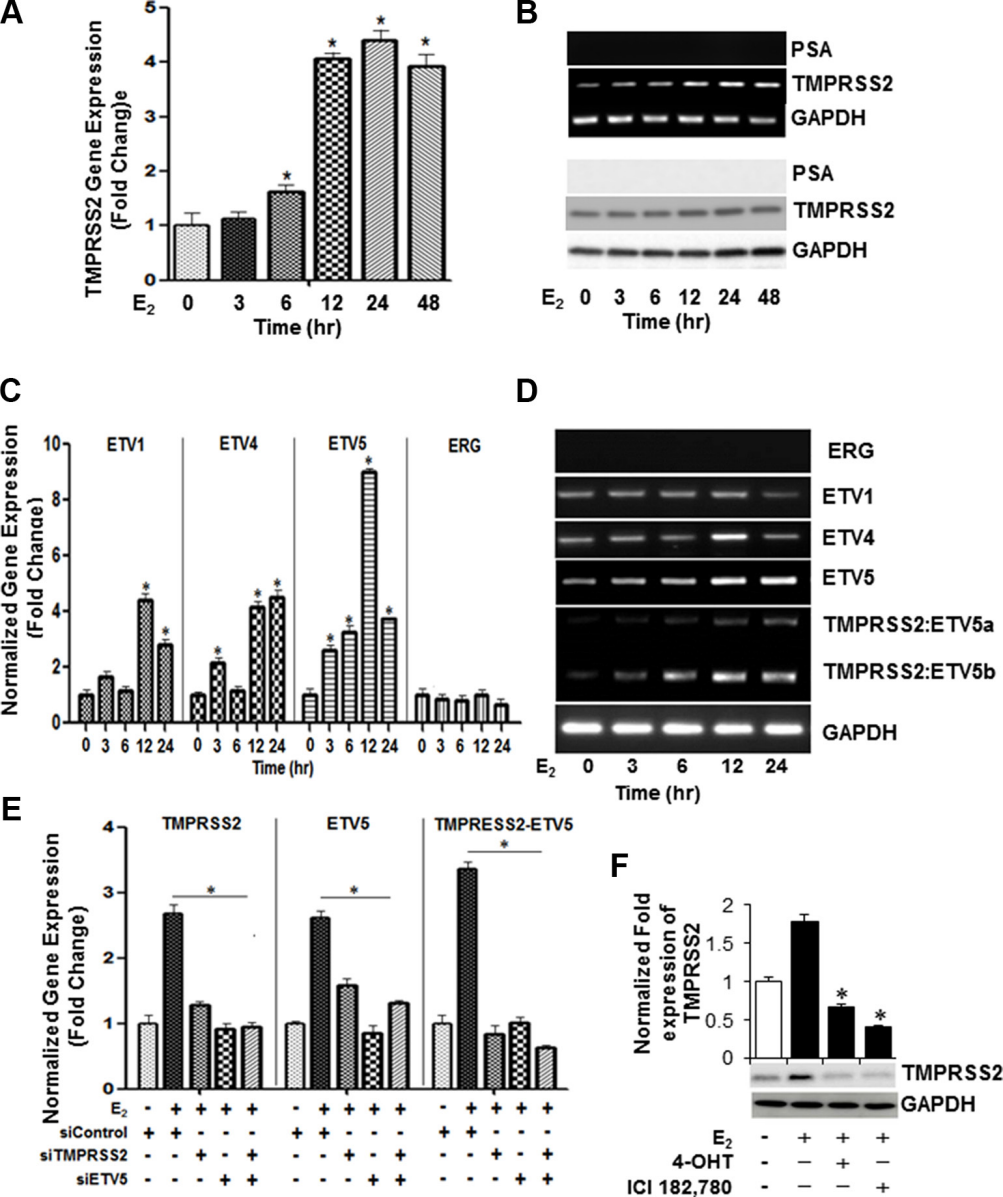
Selective expression of *ETV4, ETV5, TMPRSS2:ETV5a* and *TMPRSS2:ETV5b* transcripts in E_2_-stimulated PC-3 cells (**A**, **B**) E_2_-mediated transcriptional upregulation of *TMPRSS2* peaks at 12 hours. PC-3 cells were treated with E_2_ (10 nM) at indicated time intervals and cell lysates were examined for *TMPRSS2*, *PSA* and *GAPDH* mRNA expression by qRT-PCR (A) and conventional RT-PCR (B; upper three panels) and their protein expression by Western blot analysis (B; lower three panels). (**C**) Gene expression of *ETV1*, *ETV4*, *ETV5* and *ERG* in PC-3 cells treated with E_2_ (10 nm) for different time intervals were analyzed by qRT-PCR. GAPDH was used as an internal control for normalization of expression values. (**D**) PCR analysis reveals selective upregulation of *ETV4*, *ETV5*, *TMPRSS2:ETV5a and TMPRSS2:ETV5b* transcripts in PC-3 cells 12 hours following stimulation with 10 nM E_2_. (**E**) PC-3 cells were subjected to siRNA knockdown of *TMPRSS2*, *ETV5* or both genes after which the cells were treated with 10 nM E_2_ for 12 hours and gene expression was assessed by qRT-PCR. Data was normalized to GAPDH. (**F**) TMPRSS2 and GAPDH protein levels were measured by immunoblot analysis in PC-3 cells treated with 10 nM E_2_ in the presence or absence of ICI 182,780, or 4-OHT for 24 hours. Bar graphs represent mean ± SEM values in triplicates. * denotes significant difference compared to controls at *p* < 0.05 (*n* = 3).

Next, we examined if E_2_ growth stimulation of ERβ2-expressing PC-3 cells is associated with upregulation of androgen-regulated genes. As evidenced by selective inhibitors, E_2_ induced *TMPRSS2* gene expression (Figure 2D, left panel) following induction of NF-κB activation (Figure 2D, right panel) in PC-3 cells. As shown in Figure 2E, *TMPRSS2* gene expression increased within 24 h upon stimulation of the androgen-dependent LNCaP cells with DHT, but not with E_2_. In contrast, *TMPRSS* transcript levels increased in response to DHT (∼6-fold) or E_2_ (∼ 4-fold) in its isogenic CRPC (C4-2B) cells maintained under HDC, compared to vehicle-treated cells when measured by quantitative RT-PCR (Figure 2F; upper panel) and Western blot (Figure 2F; lower panel) analyses. In PC-3 cells, E_2_, but not DHT, increased *TMPRSS2* transcripts (Figure 2G; upper panel) and protein (Figure 2G; lower panel). Time-course experiments showed that E_2_ induces *TMPRSS2* gene expression in PC-3 cells in a time-dependent manner, which peaked (4-fold) at 12 h and sustained for 48 h (Figure 3A). The results were corroborated by conventional PCR (Figure 3B, upper panel) and immunoblot (Figure 3B, lower panel) analyses. siRNA silencing of ERα E_2_ did not inhibit the E_2_-mediated induction of TMPRSS2 protein expression in PC-3 cells (Supplementary Figure S3), thus further corroborating the direct role for E_2_-ERβ2 axis in regulating expression of TMPRSS2 in this cell line.

**Figure 4:**
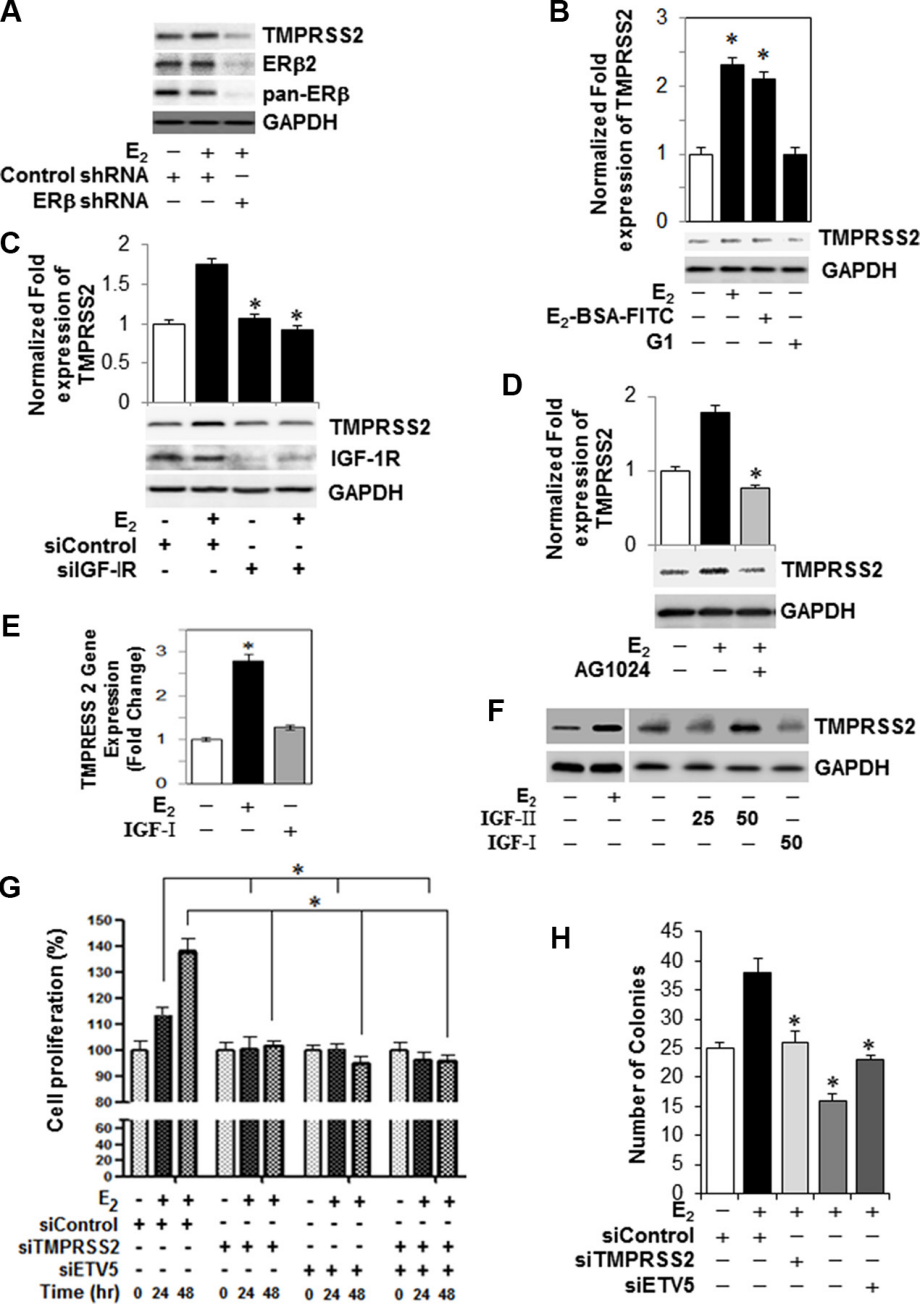
*TMPRSS2, ETVs* and *TMPRSS2:ETV5* gene fusions are involved in an IGF-1R-dependent growth stimulatory effect of E_2_-ERβ2 axis in PC-3 cells (**A**) PC-3 cells were subjected to ERβ knockdown with shRNA, and then treated with 10 nM E_2_ for 24 hours. Protein extracts were subjected to immunoblot analysis with antibodies against TMPRSS2, ERβ, ERβ2, or GAPDH. (**B**) PC-3 cells were incubated with 10 nM E_2_, 10 nM *E*_2_-BSA-FITC, or 10 nM of G-1, an agonist of the G protein-coupled estrogen receptor-1 (GPER-1) for 12 hours and *TMPRSS2* and *GAPDH* mRNAs and protein levels were quantified by qRT-PCR (upper panel) and immunoblot analysis (lower panel). (**C**) Protein levels of TMPRSS2, IGF-1R or GAPDH were measured by Western blot in PC-3 cells transiently transfected with siRNA targeting *IGF-IR* siRNA or non-targeting control siRNA and then treated vehicle or 10 nM E_2_ for 24 hours. (**D**) Assessment of TMPRSS2 protein expression in PC-3 cells treated with 10 nM E_2_ in presence or absence of 5 μM of an IGF-1R inhibitor (AG1024). GAPDH protein expression was used as loading control. (**E**, **F**) *TMPRSS2* transcripts quantified by qRT-PCR (E) and protein levels (F) as measured by Western blot analysis relative to their respective controls in PC-3 cells treated with vehicle, 10 nM E_2_, 25 ng and 50 ng IGF-I or 50 ng IGF-II for 24 hours. (**G**) PC-3 cells were transiently transfected with non-targeting control siRNA or siRNAs targeting *TMPRSS2, ETV5* or both and then treated with vehicle or 10 nM E_2_ for 0, 24 or 48 hr. Cell proliferation was assessed by Cell Counting Kit-8. Data represent Mean ± SEM in triplicates. (**H**) Colony formation assay by PC-3 cells transiently transfected with non-targeting control siRNA or siRNAs targeting *TMPRSS2, ETV5* or both and then treated with vehicle or 10 nM E_2_. Bar graphs represent mean ± SEM values in triplicates. * denotes significant difference compared to controls at *P* < 0.05 (*n* = 3).

A unique fusion between the prostate-specific, 5′-untranslational region of *TMPRSS2* and the *ETS* family *ERG*, *ETV1*, *ETV4*, *or ETV5* has been described in PC [10]. Given that the cellular signaling mechanisms of the TMPRSS2 fusions and its associated partners in PC cells are not fully understood, we sought to investigate their expression and mechanistic roles in the E_2_ growth stimulatory response in PC-3 cells. Besides *TMPRSS2*, E_2_ triggered transcriptional upregulation of *ETV5* (∼ 9-fold), ETV1 (∼ 4-fold) and ETV4 (∼ 4-fold) within 12 h in PC-3 cells (Figure 3C). Notably, the basal mRNA levels of ERG did not change in response to E_2_ (Figure 3C). Importantly, the transcriptional upregulation of *TMPRSS2* and *ETV5* was coupled with upregulation of *TMPRSS2*:*ETV5b* and, to a lesser extent, of *TMPRSS2*:*ETV5a* fusion transcripts in E_2_-treated PC-3 cells (Figure 3D). To confirm transcriptional upregulation of *TMPRSS2* gene fusions, we conducted qRT-PCR analysis of *TMPRSS2*, *ETV5* or *TMPRSS2:ETV5* fusion transcripts in E_2_-stimulated PC-3s transfected with *TMPRSS2* and/or *ETV5 siRNAs*. As shown in Figure 3E, siRNA knockdown of *TMPRSS2* and/or *ETV5* not only decreased their individual transcripts, but also *TMPRSS2*:*ETV5* gene fusions, suggesting that gene fusions are predominantly induced in E_2_-stimulated PC-3 cells. The marked suppression of *TMPRSS2* transcription by 4-OHT and IC1 182,780 attests to the selective induction of *TMPRSS2* and *ETVs* and their gene fusions by E_2_ in PC-3 cells (Figure 3F). The E_2_-mediated induction of TMPRSS2 and ERβ2 protein levels was inhibited in ERβ shRNA-silenced PC-3 cells, thus further supporting a direct role for E_2_-ERβ2 axis in regulating transcription of *TMPRSS2* and its *ETV5* fusions in this cell line (Figure 4A).

### Src-mediated activation of IGF-1R, but not GPER1, is required for E_2_-ERβ2 induced transcriptional upregulation of *TMPRSS2* gene fusions in PC-3 cells

We examined if induction of *TMPRSS2* by E_2_-ERβ2 axis is mediated through Src-activated IGF-1R or G protein-coupled estrogen receptor-1 (GPER-1), an integral plasma membrane protein with high affinity for E_2_. As shown in Figure 4B, both E_2_ and E_2_-BSA-FITC are comparable in their ability to induce *TMPRSS2* gene expression in PC-3 cells. In contrast, treatment of PC-3 cells with various concentrations of the *GPER*-1 agonist (G-1) did not induce *TMPRSS2* gene expression (Figure 4B) or protein levels (Supplementary Figure S4), suggesting that alternative pathways are involved. Notably, TMPRSS2 protein levels were significantly suppressed in IGF-1R siRNA-silenced PC-3 cells treated with E_2_, indicating that Src-activated IGF-1R is involved in TMPRSS2 protein expression in E_2_ stimualted cells (Figure 4C). This finding was further strengthened by the fact that an IGF-1R selective inhibitor, AG1024, significantly blocked E_2_-induced TMPRSS2 protein expression (Figure 4D). The E_2_-induced *TMPRSS2* gene expression through activation of IGF-1R prompted us to examine its transcriptional regulatory effect upon canonical activation by IGF-I or IGF-II in PC-3 cells. Stimulation of PC-3 cells with IGF-1 had no effect on *TMPRSS2* basal transcript and protein levels, as evidenced by qRT-PCR (Figure 4E) and immunoblot (Figure 4F) analyses. Conversely, an increase in TMPRSS2 protein levels was noted in IGF-II-treated PC-3 cells (Figure 4F). Importantly, transcriptional upregulation of *TMPRSS2*, *ETV5* and *TMPRSS2*:*ETVa/b* gene fusions were detected in IGF-II-treated PC-3 cells (Supplementary Figure S5), suggesting that IGF-II may trigger growth stimulation in PC-3 cells in a *TMPRSS2*-dependent mechanism. Together, the results implicate non-canonical Src-mediated activation of IGF-1R in the E_2_-induced expression of *TMPRSS2* and its *ETV* gene fusions in PC-3 cells.

### *ETV5* and *TMPRSS2-ETV5* gene fusions prime E_2_-ERβ-mediated growth and migration of PC-3 cells through NF-κB-dependent expression of cyclin D1 and MMPs in CRPC cells

**Figure 5:**
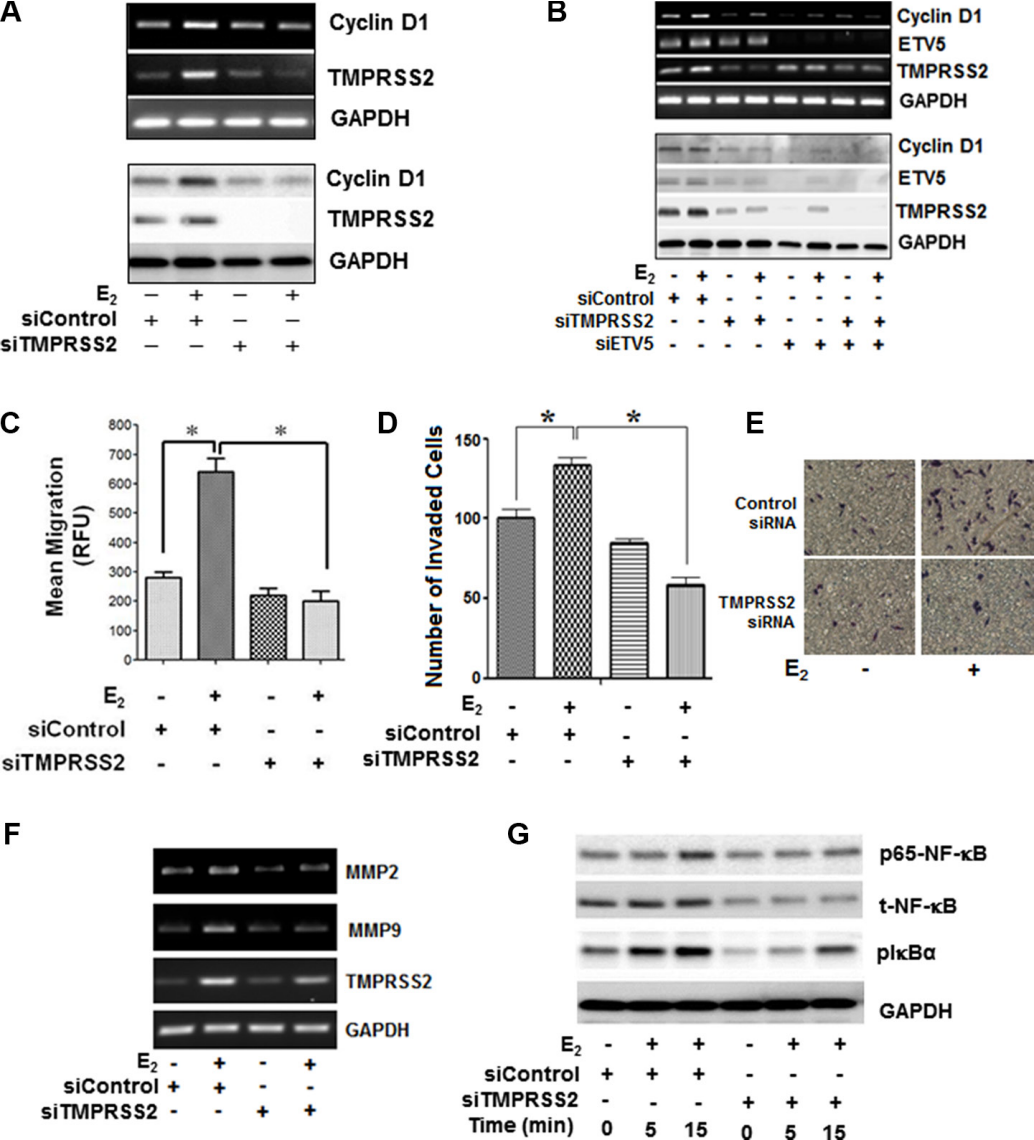
*ETV5* and *TMPRSS2-ETV5* gene fusions prime E_2_-ERβ2 mediated growth and migration of PC-3 cells through NF-κB dependent expression of cyclin D1 and MMPs in PC-3 cells (**A**) PC-3 cells were transiently transfected with siRNA targeting *TMPRSS2* or non-targeting control siRNA control, and then treated with vehicle or 10 nM E_2_ for 12 or 24 hours. Transcripts and protein levels of cyclin D1, TMPRSS2 and GAPDH were measured by RT-PCR (upper three panels) and immunoblot (lower three panels) analyses. (**B**) Transcript and protein levels *cyclin D1*, *TMPRSS2* and *ETV5* were examined by RT-PCR (upper three panels) and Western blot (lower three panels) in PC-3 cells transfected with siRNA targeting *TMPRSS2* or non-targeting control siRNA, and then treated with vehicle or 10 nM E_2_ for 12 or 24 hours, respectively. A transwell migration assay was performed to assess the number of PC-3 cells migration. The cells were transiently transfected with siRNA targeting *TMPRSS2* or non-targeting control siRNA control and then treated with vehicle or E_2_ (10 nM) and allowed to migrate to the bottom wells through a 4-μm pore membranes for 24 hours. (C–E). Migrated cells were stained with Calcein-AM uptake (**C**) or Diff-Quick method (**D**) or methylene blue (**E**, **F**) *MMP2* and *MMP9* gene expression in PC-3 cells transiently transfected with siRNA targeting *TMPRSS2* or non-targeting control siRNA, and then treated with 10 nM E_2_ or vehicle for 12 hours. (**G**) PC-3 cells transfected with siRNA targeting *TMPRSS2* or non-targeting control siRNA, and then treated with vehicle or 10 nM E_2_ for 24 hours. Cell lysates were subjected for immunoblot analysis with antibodies against NF-κB, pNF-κB (p65), pIkB, or GAPDH. Bar graphs represent mean ± SEM values in triplicates. * denotes significant difference compared to controls at *P* < 0.05 (*n* = 3).

Next, we investigated if *TMPRSS2, ETV5*, or *TMPRSS2-ETV5* gene fusions are involved in E_2_-ERβ axis induction of mitogenic response and migration of PC-3 cells. As shown in Figure 4G, siRNA silencing of *TMPRSS2* and/or *ETV5* significantly inhibited E_2_ induced PC-3 cell proliferation at 24 h and 48 h compared to vehicle-treated or control siRNA transfected cells. Likewise, silencing of *TMPRSS2* and/or *ETV5* genes suppressed the ability of E_2_-stimulated PC-3 cells to form colonies, further attesting to the potential involvement of *ETV5* and *TMPRSS2-ETV5* fusions in E_2_-ERβ mediated PC-3 cell growth (Figure 4H). Next, we examined if cyclin D1 is a direct transcriptional target for *ETV5* and *TMPRSS2-ETV5* gene fusions in PC-3 cells. Figure 5A and 5B show siRNA silencing of *TMPRESS2* and/or *ETV5* genes primed down-regulation of E_2_-ERβ induced cyclin D1 gene (upper panels) and protein levels (lower panels), thus reinforcing the potential role of cyclin D1 in mediating *ETV5* and/or *TMPRSS2-ETV5* gene mitogenic response of E_2_ in PC-3 cells. Moreover, we used Boyden chamber assays to examine the effect of *ETV5* and *TMPRSS2-ETV5* gene fusions on invasion and migration of PC-3 cells. The number of migrated (Figure 5C) and invaded (Figure 5D and 5E) PC-3 cells were significantly reduced in *TMPRSS2* siRNA-transfected E_2_-treated PC-3 cells, potentially through down-regulation of MMP2 and MMP9 (Figure 5F). Since NF-κB is implicated in their regulation, we examined if it plays a pivotal role in mediating the expression of MMPs by *TMPRSS2* and *TMPRSS2* gene fusions in the E_2_-stimulated PC-3 cells. Figure 5G shows that E_2_ activates NF-κB within 15 min in PC-3 cells, and that *TMPRSS2* is required for its activation. The results not only implicate a positive activation loop between these factors, but also the potential involvement of NF-κB in *TMPRSS2*-mediated *MMP* gene expression in E_2_-treated PC-3 cells. Together, the results suggest TMPRSS2 is a critical player in promoting E_2_-mediated stimulation of growth and migration of PC-3 cells, primarily through NF-κB activation.

## DISCUSSION

Understanding the underlying molecular pathways that lead to the development of CRPC may establish a more rational approach for development of new therapeutic strategies. Despite the historical use of estrogen in the treatment of PC, little is known about its direct role in prostate tumorigenesis. This is particularly important since circulating estrogen-to-androgen ratio increases with age [14], a risk factor for PC development and progression. In addition, intracrine estrogen synthesis and metabolism in the tumor microenvironment have been implicated as the driving force of PC outgrowth following androgen ablative therapy [15]. This notion was strengthened by the fact that the level of aromatase expression (CYP19A1), the enzyme that catalyzes conversion of testosterone into estradiol, is elevated by 30-fold in metastatic sites compared with primary prostate tumors [16], suggesting that estradiol may contribute to disease progression. To our knowledge, the present study provides the first molecular link between E_2_ and CRPC cell growth *in vitro*.

The functional significance of ERβ in prostate tumorigenesis remains a matter of conjecture. Unlike ERα, epidemiological and tissue expression profile studies demonstrated the anti-oncogenic and protective roles ERβ play in the prostate [17]. In contrast, recent evidence revealed that activation of ERβ by genistein promotes, and inhibition of ERβ by ICI 182, 780 suppresses metastatic PC progression in a xenograft model system [18]. We have recently demonstrated selective expression of ERβ in patients with advanced PC [6]. Nuclear ERβ expression was detected with varying degree in 100% of osseous and non-osseous metastatic PC samples, suggesting it plays a role in disease progression [6, 19]. However, the underlying mechanisms by which ERβ splice variants promote disease progression remain elusive, especially under hormone deprivation conditions. The selective growth stimulation by E_2_ in PC-3 cells, but not DU-145 cells implicates ERβ2, but not ERβ1, in E_2-_ induced mitogenic response in this cell line. The co-localization of ERβ2 with IGR-1R and its co-precipitation with IGR-1R and Src further corroborate its crucial role in priming proliferative and migratory responses via Src-IGF-IR axis in PC-3 cells. Consistent with our findings, ER-expressing undifferentiated prostaspheres have been shown to exhibit proliferative response to E_2_ [20]. High constitutive expression of ERβ subtype compared to ERα in CRPC cells, such as PC-3 and C4-2B, may explain activation and a potential contributory role of E_2_-ERβ signaling axis in growth and clonal expansion of these cells. Our findings suggest that a subset of ERβ-expressing PC cells may be direct estrogen targets in patients following androgen ablation therapy. Whether this effect is mediated by circulating or residual estrogens certainly warrants further investigation.

TMPRSS2, a type II transmembrane member of the serine protease family, is implicated in PC progression [9] by modulating the vascular function and angiogenesis [21]. However, the regulatory mechanisms involved in expression of *TMPRSS2* fusions in PC cells remain largely unknown. We report herein, for the first time, that *TMPRSS2* is expressed in response to E_2_ in both AR-expressing and AR-null CRPC cells under ADC. The E_2_-induced expression of *TMPRSS2* mediates PC-3 cell growth through non-canonical activation of ERβ under ADC. We have shown that IGF-1R inhibition blocked the expression of *TMPRSS2* in E_2_-stimulated PC-3 cells by AG1024 (IGF-1R inhibitor). Additionally, silencing IGF-1R in PC-3 cells decreased TMPRSS2 expression and inhibited E_2_ or IGF-I mediated PC-3 cell proliferation. Dysregulation of various components of the IGF-1R system has been reported at different tumor stages and has been implicated in tumorigenesis through non-canonical pathways [5]. Recent studies revealed molecular pathways by which IGF-II ignites the *de novo* steroidogenesis engine, confers androgen independent growth in PC cells [22], and promotes molecular events associated with PC progression to castration resistance [23, 24]. However, the underlying mechanisms of IGF-II remain largely uncharacterized. Our study demonstrates that IGF-II, but not IGF-I, promotes TMPRSS2 protein expression. The finding suggests that IGF-II may promote development and progression of PC though transcriptional upregulation of androgen-dependent genes, such as TMPRSS2. Therefore, the direct relationship between IGF-1R, c-Src and ERβ2 is a novel observation and shows that activation of this complex is required for or E_2_-BSA-FITC or E_2_ mediated-regulation of *TMPRSS2* in AR-null PC cells.

A complex array of chromosomal rearrangements causes the fusion of *TMPRSS2* to *ETV* genes, leading to the generation of a wide variety of C-terminally truncated *TMPRSS2* fused to N-terminally truncated *ERG/ETV*. In PC, *TMPRSS2* also exists as gene fusion with erythroblastosis virus E26 (ETS) gene family members. The ETS are oncogenic transcription factors encompassing highly conserved DNA binding and an N-terminal regulatory domains. In all cases, ETS factors are known to act as positive or negative regulators of the gene expression that are involved in various biological processes, including cellular proliferation, tissue remodeling, angiogenesis, transformation and metastasis [9]. *TMPRSS2* gene fusion with *ETV1, ETV4 or ETV5* has also been documented in PC [25]. In the present study, the time-dependent transcriptional upregulation of *TMPRSS2* by E_2_ is concomitantly associated with expression of ETV family (*ETV1, ETV4* and *ETV5*) and *TMPRSS2:ETV5a* and *TMPRSS2:ETV5b* gene fusions in PC-3 cells, presumably through induced TMPRSS2 expression. We also showed that TMPRSS2 and TMPRSS2:ETV5 fusions have growth-promoting activity and increase colony formation in the presence of E_2_ stimulation in the AR-null PC-3 cell lines. Conversely, *TMPRSS2:ETV5* knockdown suppressed cell proliferation and metastases as evidenced by decreased cyclin D1 and MMP expression, as well as a decrease in colony formation by E_2_ stimulation in PC-3 cells. The *ETV5* fuses with the 5′ untranslated region of *TMPRSS2* in human PC tissue samples and induces cell invasion. The *TMPRSS2*:*ETV5a* transcript contained exon 1 of *TMPRSS2* fused to exon 2 of *ETV5* whereas *TMPRSS2*:*ETV5b* contained exons 1 to 3 of *TMPRSS2* fused to exon 2 of *ETV5* [26]. A recent study demonstrates that *TMPRSS2/ERG(T/E)* fusion gene can promote PC invasion and, to a lesser extent, proliferation and decrease differentiation via activation of c-myc, uPA, and MMPs, all of which have been previously implicated in PC initiation and progression [27]. Taken together, the results support the hypothesis that *TMPRSS2-ETV* fusions may be the driving force of the growth-promoting ability of E_2_-ERβ axis in CRPC cells, suggesting it may contribute to disease progression in PC patients through clonal expansion of castration-resistant phenotype. Thus, therapies targeted towards this axis may offer an attractive option for disease management in CRPC patients.

The transcriptional upregulation of *TMPRSS2-ETV5* fusions by E_2_ through ERβ-Src-IGF-1R complex was associated with NF-κB activation in PC-3 cells. The NF-κB pathway activation is associated with aggressive clinical behavior in PC. Both clinical and preclinical observations have shown that NF-κB plays an important role in PC growth, survival, metastatic progression, angiogenesis, and tumorigenesis. NF-κB transcriptional activity is increased by *TMPRSS2-ERG* fusions through toll-like receptors [28]. However, our results indicate that the upregulation of *TMPRSS2-ETV gene* fusion by E_2_ enhances the tumor-promoting activities of the NF-κB pathway through induction of growth and metastasis-related genes (cyclin D1 and MMPs). These findings provide further evidence of a relationship between E_2_, ERβ, c-Src and IGF-1R in PC cells and delineate the molecular mechanisms that underlie expression of *TMPRSS2-ETV5* fusions in AR-null PC cells. Taken together, our findings strongly indicate that *TMPRSS2:ETV5* is not only an estrogen-responsive gene, but also a pivotal player in the outgrowth of castration-adapted PC cells under HDC. However, further investigations are required to decipher the underlying mechanisms by which *TMPRSS2* or *TMPRSS2:ETV5* fusion promotes growth, colony formation, and migration of E_2_-stimulated AR-null CRPC cells.

**Figure 6:**
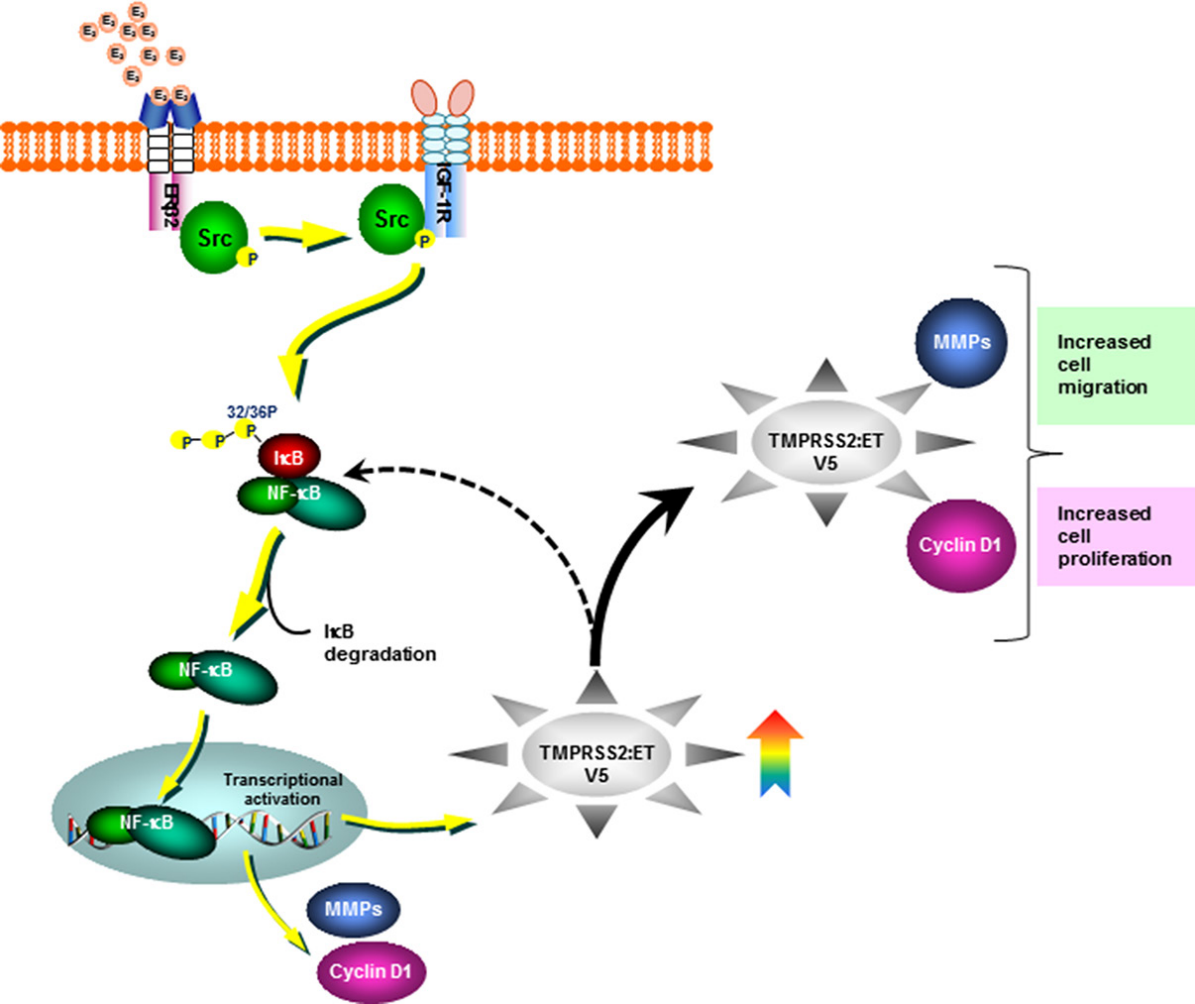
Schematic representation depicting Src-IGF-1R dependent non-canonical activation of ERβ2 by estradiol (E_2_) The activation of E_2_-ERβ2 axis leads to expression of the androgen-dependent TMPRSS2-ETV5a and b gene fusions through IGF-1R dependent-activation of NF-κB in the CRPC AR-null PC-3 cells.

Collectively, our novel findings unravel the underlying mechanisms that govern the functional significance and the molecular link between the oncogenic axis of ERβ2-IGF-1R-*TMPRSS2:ETV5* fusion in mediating E_2_-induced growth and migration of AR-null PC-3 cells (Figure 6). Selective targeting of such critical players may have significant preventive and therapeutic implications for CRPC.

## MATERIALS AND METHODS

### Reagents

Penicillin, streptomycin, charcoal-stripped fetal bovine serum (CS-FBS), and fetal bovine serum (FBS), were purchased from Invitrogen (Camarillo, CA, USA). The agonist G-1 of the G protein-coupled estrogen receptor-1 (GPER-1) and the ER antagonist ICI 182,780 were from TOCRIS (Bristol, UK); E_2_, 5α-Dihydrotestosterone (DHT), or E_2_-BSA-FITC, 4-hydroxytamoxifen (4-OHT) were obtained from Sigma (St. Louis, MO, USA). The recombinant human IGF-I was from R&D Systems (Minneapolis, MN, USA) and the recombinant human IFG-II was from PerproTech (Rocky Hill, NJ, USA). The selective inhibitors of the Src family of protein tyrosine kinases (PP2) and IGF-1R (AG1024) were from EMD Millipore (Calbiochem, Temecula, CA). The NF-κB activation inhibitor II was purchased from EMD Millipore. Unless otherwise indicated, all other drugs were obtained from Sigma-Aldrich (St. Louis, MO, USA).

### Cell lines

Metastatic castration-resistant C4-2B (a kind gift from Dr. Leland Chung, Cedar-Sinai Medical Center, Los Angeles, CA), androgen-independent PC-3 prostate adenocarcinoma cell, and the human androgen-dependent prostate cancer LNCaP epithelial cells (ATCC, Manassas, VA USA), were cultured in RPMI1640 media supplemented with 10% fetal bovine serum, 2 mM L-glutamine and 1% penicillin/streptomycin (Invitrogen). The PrEC cells (ATCC) were maintained in prostate epithelial growth media (PrEBM) containing supplements and growth factors (BPE, hydrocortisone, hEGF, epinephrine, insulin, triiodothyronine, transferrin, gentamicin/amphotericin B, and retinoic acid) from Cambrex Bio Science (Walkersville, MD, USA). For routine maintenance, each cell line was grown as a monolayer at 37°C in a water-saturated atmosphere of 20% oxygen, 5% CO_2_. All cell lines were characterized and authenticated by the supplier (ATCC) using short tandem repeat (STR) profiling. Unless otherwise indicated, estradiol (E_2_) experiments were carried out in cells cultured in phenol-red free media supplemented with 10% charcoal-stripped FBS (Invitrogen). Supplementary Figure S7 provides the certificate of analysis for the authentication of PC-3 cells from ATCC.

### Transfection

Before treatment with E_2_, the medium was changed to RPMI supplemented with 10% CS-FBS. PC-3 cells were transiently transfected with control non-targeting siRNA or specific siRNAs targeting *TMPRSS2*, *IGF-1R*, or *ETVs* (Santa Cruz Biotechnology, CA, USA). In another set of experiments, PC-3 cells were transfected with a control shRNA plasmid, shRNA targeting *ERβ* or *ERα* using Lipofectamine 2000, according to the manufacturer's instructions (Invitrogen).

### Cell proliferation assay

The assay was performed as we described previously [5]. Briefly, cells were seeded in a 96-well plate and treated with vehicle (alcohol) or E_2_ 10 nM in triplicates. After 24 or 48 hours of incubation, the medium was replaced with fresh medium containing 0.5 mg/ml MTT [3-(4,5-dimethylthiazol-2-yl)-2,5-diphenyltetrazolium bromide] or the Cell Counting Kit-8 (CCK-8; Donjindo Molecular Technologies, Rockville, Maryland, USA). After 4 hours, the supernatants were removed, and the resulting formazan crystals were measured spectrophotometrically using a microplate reader (Corning, NY, USA).

### Clonogenic survival assay

PC-3 cells were trypsinized and plated in triplicate into 60 mm at a density of 250 cells per well in 10 mm dishes. The cells were then transfected with control siRNA or siRNA targeting *TMPRSS2, ETV5* or both for 24 h and allowed to incubate for 12 days in growth media. The cells were washed and stained with crystal violet, and the colonies containing > 50 cells were counted. Plating efficiency was calculated by dividing the average number of cell colonies per well by the amount of cells plated. Survival fractions were calculated by normalization to the plating efficiency of appropriate control groups.

### PCR and immunoblot analyses

Oligonucleotide primers were synthesized by Integrated DNA Technologies and are listed in Table 1. RNA was subjected to conventional and quantitative RT-PCR analyses using selective primer sequences as we described previously [25]. The universal ERβ PCR amplimer set amplifies wild-type and all subtypes. Protein extracts were subjected to immunoblot analysis with antibodies to p-Src, c-Src (32G6), p-IGF-ΙR, IGF-ΙR, cyclin D1, p-p65 NF-κB, NF-κB, IκB, and p-IκB (Cell Signaling Technology); PSA, ETV5, or GAPDH (Santa Cruz Biotechnology); ERβ; or *TMPRSS2* (Abcam, Cambridge, MA, USA). Immune complexes were detected with appropriate secondary antibodies and chemiluminescence reagents (Pierce Biotechnology, Rockford, IL, USA). Densitometry of immunoblot signals was quantified using the VersaDoc^™^ imaging system (Bio-Rad Laboratories, Hercules, CA, USA).

**Table 1:** Primer sequences used for RT-PCR

quantitative RT-PCR		
Primer name	Forward primer sequence	Reverse primer sequence
ETV1	5′-CCAAACTCAACTCATACACCGAAACC-3′	5′-GGAGGGAAGCTTTGGCTGGC-3′
ETV4	5′-CCCTACCAACACCAGCTGTC-3′	5′-GAGAAGCCCTCTGTGTGGAG-3′
ETV5	5′-CCACCTCCAACCAAGATCAAACG-3′	5′-CACCTTGAACTGGGCCAGCTG-3′
TMPRSS2	5′-GTCCCCACTGTCTACGAGGT-3′	5′-CAGACGACGGGGTTGGAAG-3′
PSA	5′-AGGCCTTCCCTGTACAC AA-3′	5′-GTCTTGGCCTGGTCATTTCC-3
IGF-1R	5′-AGGAACAACGGGGAGAGAGC-3′	5′-ACC GGTGCCAGGTTATGATG-3′
pan-AR	5-CCATCTTGTCGTCTTCGGAAATGTTATG AAGC-3′	5-AGCTTCTGGGTTGTCTCCTCAGTGG-3
pan-ERβ	5′-AGAGTCCCTGGTGTGAAGCAA-3′	5′-GA AGCGCAGAAGTGAGCATC-3′
TMPRSS2:ETV5	5′-AGGCTTCCAACCCCGTCGTC-3′	5′-ACTTGCTGATCATAAAACCCGTCCAT-3′
MMP2	5′-TGATGGTGTCTGCTGGAAAG-3′	5′-GACACGTGAAAAGTGCCTTG-3′
MMP9	5′-GGAGACCTGAGAACCAATCTC-3′	5′-TCCAATAGGTGATGTTGTGGT-3′
GADPH	5′-TCCCTGAGCTGAACGGGAAG-3′	5′-GGAGGAGTGGGTGTCGCTGT-3′
**RT-PCR**		
**Primer name**	**Forward primer sequence**	**Reverse primer sequence**
TMPRSS2:ETV5a/b	5′-CGCGAGCTAAGCAGGAGGC-3′	5′-CCGTTTGATCTTGGTTGGAGGTG-3′
ERβ2	5′-TCTCCTCCCAGCAGCAAT CC-3′	5′-GGT CAC TGC TCC ATC GTT GC-3′

### Immunofluorescence

Cells were seeded on glass chamber slides and treated as described in the figure legends. Cells cultured on glass coverslips were washed twice with PBS, pH 7.2, and fixed under permeabilized [29] or non-permeabilized, PBS containing 2% PFA, 0.05% glutaraldehyde, and 120 mM sucrose [30], conditions for 30 min at room temperature. The non-permeabilized fixative conditions preserve plasma membrane integrity and prevent antibodies from binding to endogenous cellular targets. After fixation, cells were washed in PBS and incubated at room temperature with normal goat serum to block nonspecific binding. The coverslips were incubated overnight at 4°C with primary mouse anti-IGF-1R (clone GR11; EMD Millipore) or rabbit anti-ERβ (clone NBP1-40777, abcam, Cambridge, MA) antibody or ESR2 Beta-2 Antibody (cat# 55470-1-AP; Proteintech Group, Inc., Rosemont, IL), rinsed with PBS, and incubated with the appropriate fluorescently-labeled secondary antibodies, mouse Alexa 594 (red) or 350 (blue) or rabbit Ab Texas red (Red) or 350 (blue), and then examined under a Nikon fluorescence microscope.

### Co-immunoprecipitation

Protein-protein interaction between ERβ, Src and IGF-1R was determined using Pierce Classic Magnetic IP/Co-IP Kit according to manufacturer's protocol (Thermo Fisher Scientific Inc, Rockford, IL, USA). Briefly, PC-3 cells cultured in CS-FBS-supplemented RPMI media were treated with vehicle or E_2_ (10 nM) for 5 min. Cell extracts (500 μg) were lysed with an IP lysis/wash buffer containing 0.025 M Tris, 0.15 M NaCl, 0.001 M EDTA, 1% NP40, 5% glycerol, and protease inhibitor cocktail (Roche Applied Science, Indianapolis, IN, USA), and then incubated overnight at 4°C with 5 μg of either normal rabbit or mouse IgG (EMD Millipore), rabbit anti-ERβ mAb (#sc-8974; Santa Cruz Biotechnology, Inc.; Dallas, TX), mouse anti-c-Src (32G6) mAb (#2011; Cell Signaling Technology Inc.; Danvers, MA), or mouse anti-IGF-1R mAb (#sc-462; Santa Cruz Biotechnology, Inc.). Immune complexes were pulled down by protein A/G magnetic beads (0.25 mg) followed by low-pH elution. Eluted proteins were then boiled in SDS sample buffer and subsequently fractionated by SDS-PAGE. Following semi-dry transfer, the membranes were analyzed by immunoblotting using rabbit anti-ERβ (clone NBBP1-40777, Abcam), IGF-1R (sc-713; Santa Cruz Biotechnology, Inc.), c-Src (#2123S; Cell Signaling Technology, Inc.; Danvers, MA), rabbit anti-ERβ2 (Proteintech Group, Inc., Rosemont, IL), or GAPDH (Santa Cruz Biotechnology) antibodies at recommended dilutions. Immune complexes were detected with appropriate secondary antibodies and chemiluminescence reagents (Pierce Biotechnology). Densitometry of immunoblot signals was quantified as described above.

### Cell migration

Boyden chamber assay was carried out as we described previously [25]. PC-3 cells (1 × 10^4^ cells/well) were transfected with non-targeting control or *TMPRSS2* siRNA for 24 h. Aliquots of cells were stained with Calcein-AM according to the manufacturer's protocol (Molecular Probes, Grand Island, NY). Stained and unstained cells (1 × 10^4^ cells/well) were added to the upper chamber in serum-free medium and were monitored for 24 h for migration towards the lower chamber containing serum-free medium containing 10 nM E_2_. The stained migrated cells were measured by a fluorescent plate reader (BioTek, Winooski, VT, USA) after 30 min. For the unstained cells, the non-migrated cells in the upper chamber were removed, inserts were individually stained with Diff-Quick method (International Reagents Corp., Kobe, Japan), and the migrated cells adherent to the underside of the filter were examined, counted, and photographed under a light microscope at ×200 magnification.

### Statistical analysis

Data are presented as means ± S.E.M. of more than three independent experiments performed in triplicate. In case of Western blots, a representative figure is depicted. Comparisons between multiple groups were performed with ANOVA with Bonferroni's test using GraphPad Prism (La Jolla, CA). Statistical significance was considered at *P* < 0.05.

## SUPPLEMENTARY MATERIALS FIGURES


