# Supplementary material for: Estradiol-ERβ2 signaling axis confers growth and migration of CRPC cells through TMPRSS2-ETV5 gene fusion
Source: Oncotarget. 2016 Aug 17;8(38):62820–33. doi: 10.18632/oncotarget.11355 (PMC5609883; doi:10.18632/oncotarget.11355)
Supplement: Supplementary file 1 [file oncotarget-08-62820-s001.pdf]

Estradiol-ERβ2 signaling axis confers growth and migration of CRPC cells through TMPRSS2-ETV5 gene fusion

Supplementary Materials

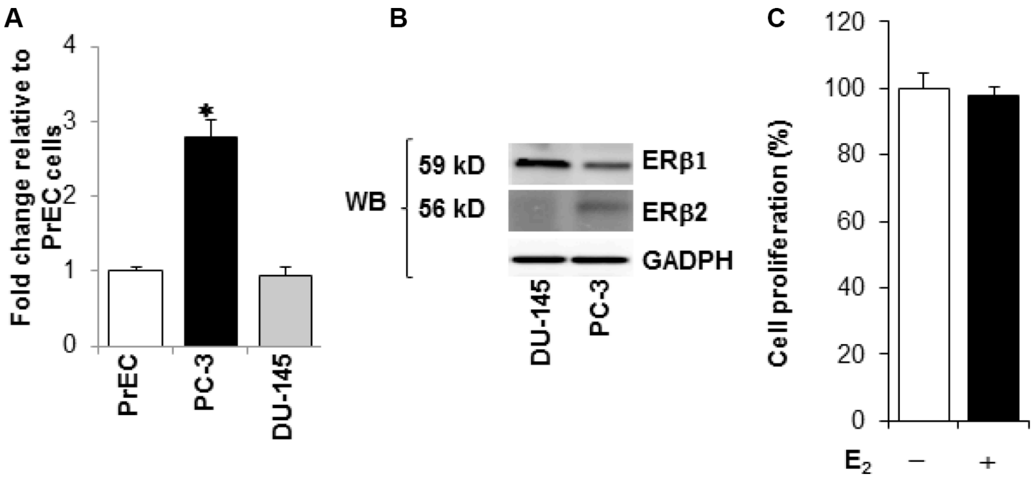

Supplementary Figure S1: ERβ1 and ERβ2 expression in PC cells. (**A**) ERβ2 gene expression was measured by qRT-PCR in PC-3, DU-145, and normal prostate epithelial cells (PrEC). The mRNA levels were expressed relative to the housekeeping gene GAPDH and presented as fold change in PC cells relative to PrEC cells. Bars represent the Mean ± SE of 3 independent replicates. Bar graphs represent mean ± SEM values in triplicates. * denotes significance at $p < 0.01$ compared to PrEC cells. (**B**) Western blot analysis of expression levels ERβ1 and ERβ2 and GAPDH in DU-145 and PC-3 cell lines. The PC cells were maintained for 24 hr in 10% CS-FBS. Then protein expression levels were assessed by Western blot analysis as described in Materials and Methods section. The representative results obtained from 3 separate experiments are shown. (**C**) DU-145 cells were subjected to 10 nM E_2 for 48 h and cell growth was assessed using the MTT assay.

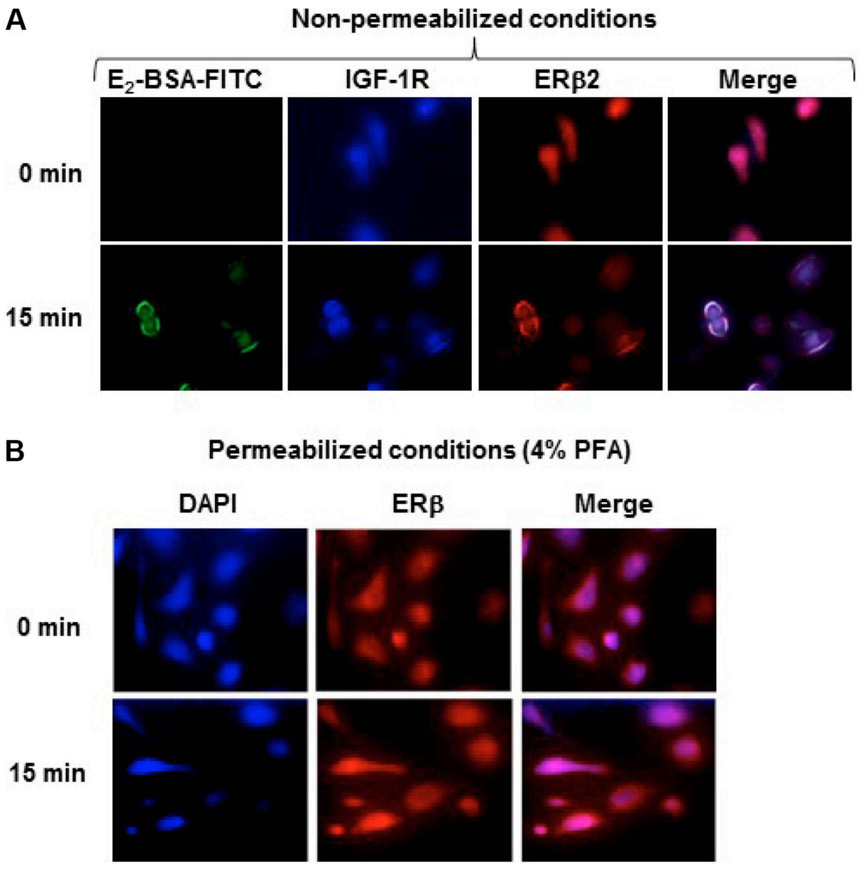

Supplementary Figure S2: Nuclear translocation and co-localization analysis of ERβ2 and IGF-1R in PC-3 cells under permeabilization and non-permeabilization conditions. (**A**) PC-3 cells cultured in chamber slides were stimulated with E_2-BSA-FITC and then fixed under non-permeabilization conditions, and subjected to immunofluorescence staining with antibodies against ERβ2 and IGF-1R. The E_2-BSA-FITC (green) stimulation triggered co-localization of IGF-IR (blue) and ERβ2 (red) on the plasma membrane of PC-3 cells as described in Materials and Methods. (**B**) Nuclear translocation of ERβ in PC-3 cells stimulated with E_2 and then fixed under permeabilization conditions. Shown is ERβ (red) colocolize with DAPI (blue) in the nuclei in PC-3 cells. The images were captured using Leica fluorescence microscope.

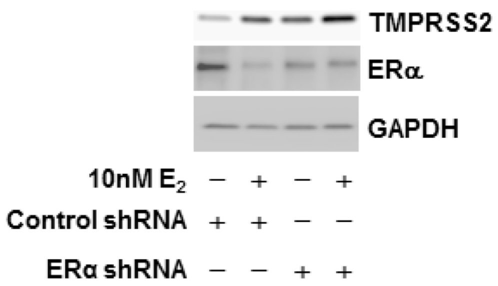

Supplementary Figure S3: TMPRSS2 protein expression in ERα-knockdown PC-3 cells. Cells transfected with ERα shRNA or control shRNA, and then treated with 10 nM E_2 for 24 hrs. Protein extracts were subjected to immunoblot analysis with antibodies against TMPRSS2, ERα, and GAPDH.

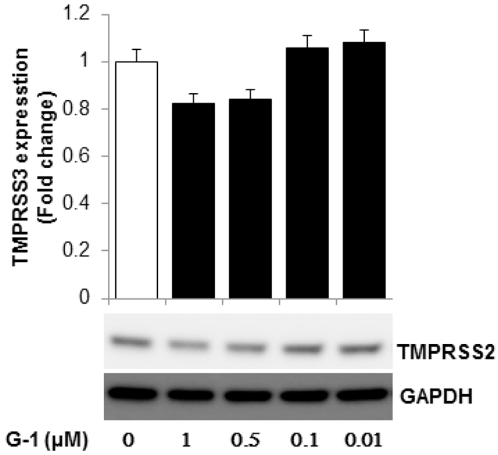

Supplementary Figure S4: Expression of TMPRSS2 in G-1 treated PC-3 cells. Lysates of PC-3 cells treated with vehicle (ethanol) or G-1 (0.01, 0.1 0.5, and 1 µM) were subjected to Western blot analysis to determine TMPRSS2, and GAPDH. Densitometry of immunoblot signals was quantified. Bar graphs represent Mean ± SEM values in triplicates (*n* = 3).

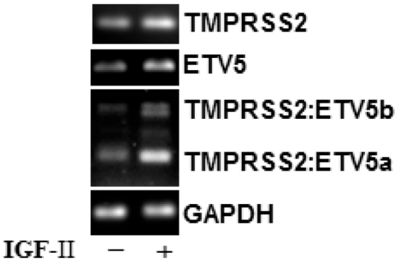

Supplementary Figure S5: Selective expression of *TMPRSS2, ETV5, TMPRSS2:ETV5a* and *TMPRSS2:ETV5b* transcripts in IGF-II -stimluated PC-3 cells. IGF-II -mediated transcriptional upregulation of *TMPRSS2* peaks at 12 hours. PC-3 cells were treated with IGF-II (50 ng) at indicated time intervals and cell lysates were examined for *TMPRSS2, ETV5, TMPRSS2:ETV5a* and *TMPRSS2:ETV5b* and GAPDH mRNA expression by RT-PCR analysis.

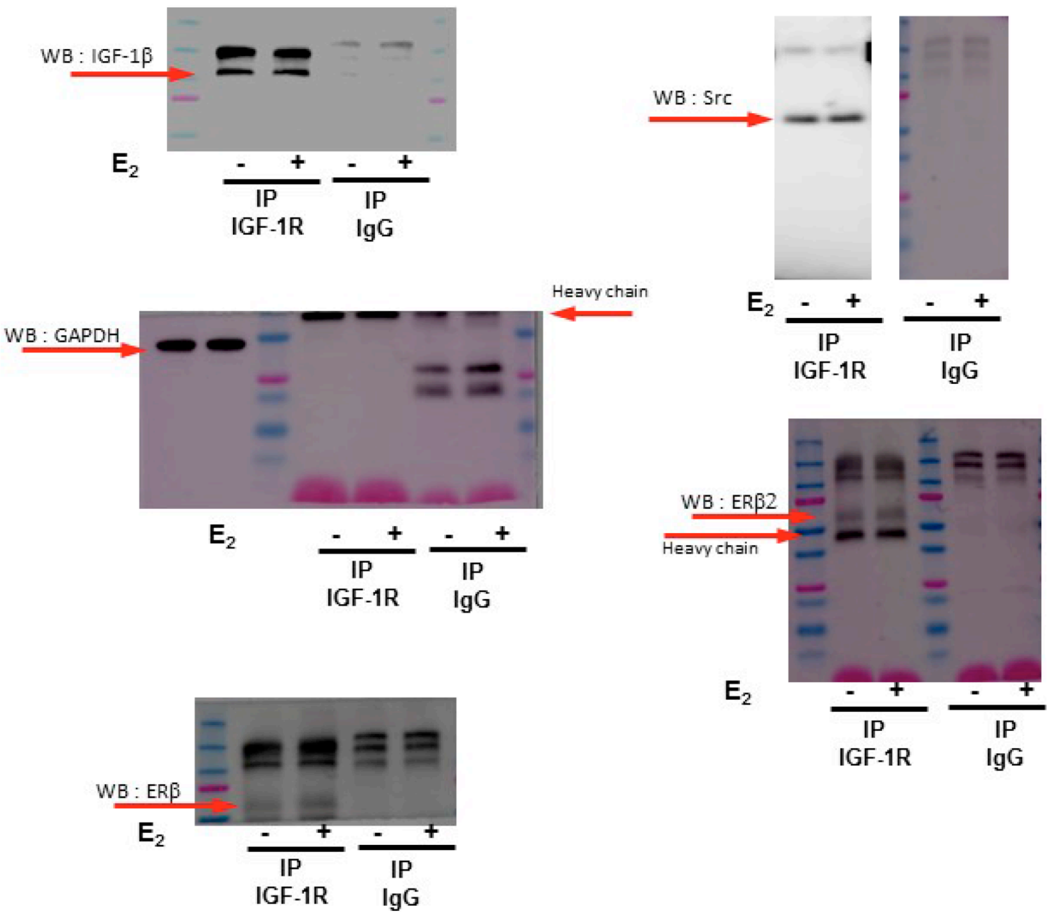

Supplementary Figure S6A: Whole membrane images for the Western blot and Co-IP experiments.

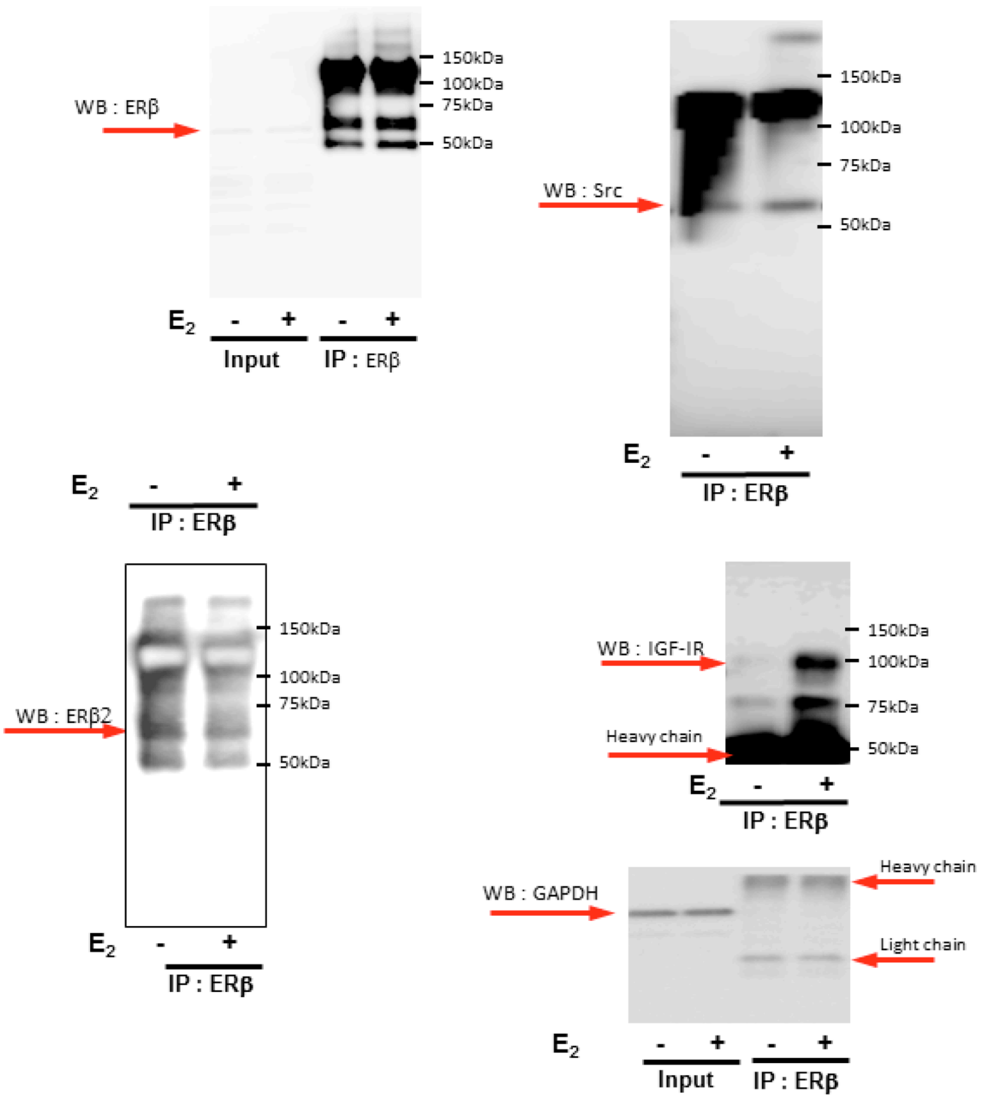

Supplementary Figure S6B: Whole membrane images for the Western blot and Co-IP experiments.

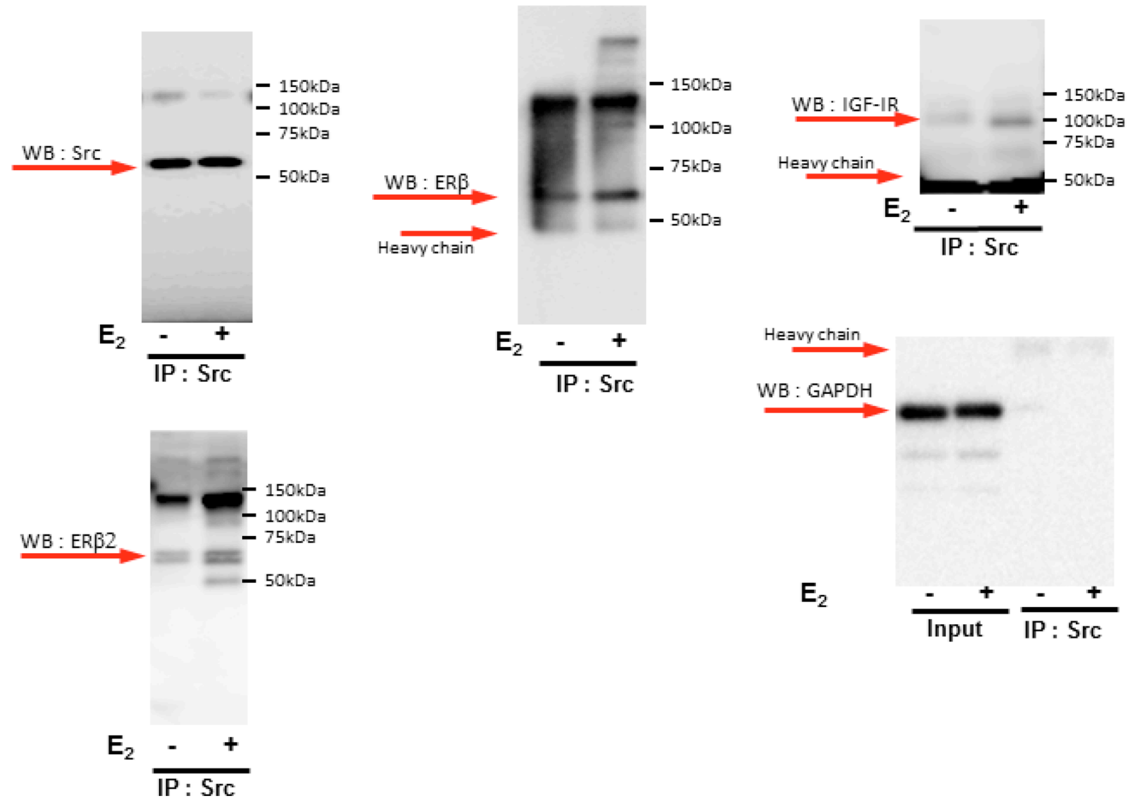

Supplementary Figure S6C: Whole membrane images for the Western blot and Co-IP experiments.

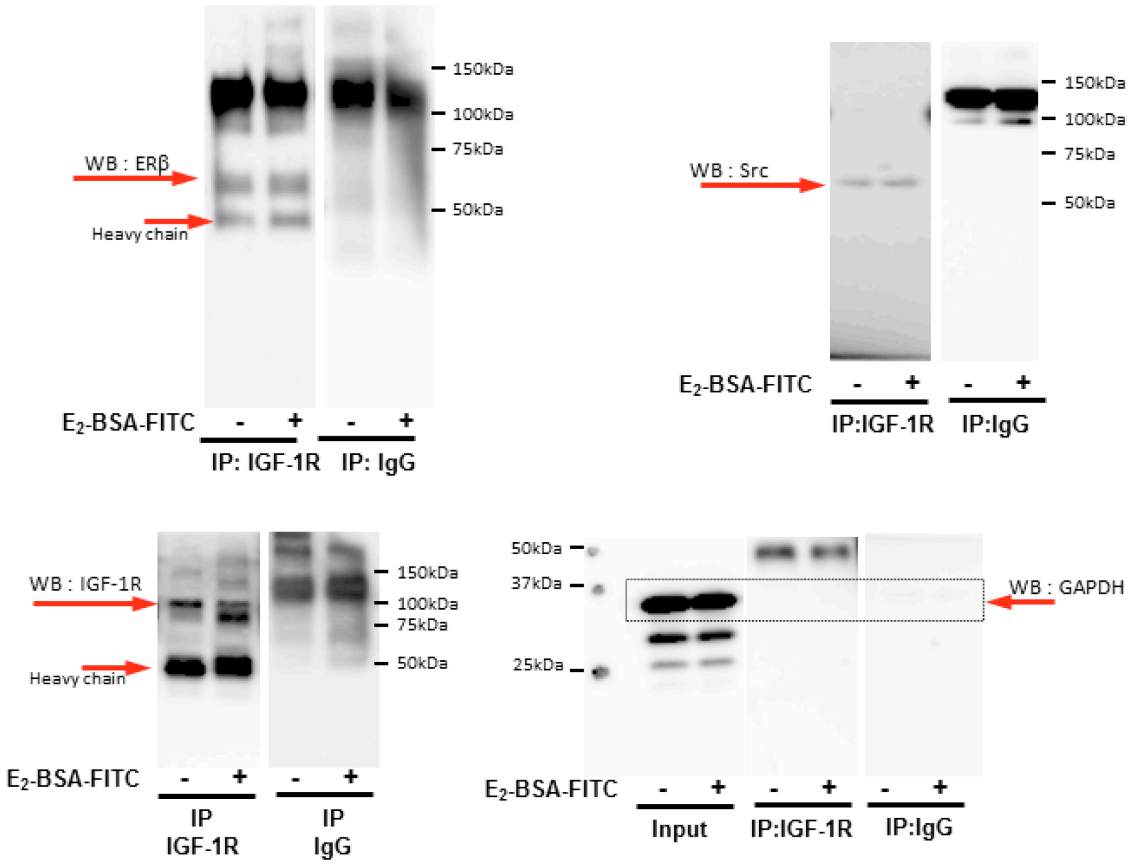

Supplementary Figure S6D: Whole membrane images for the Western blot and Co-IP experiments.

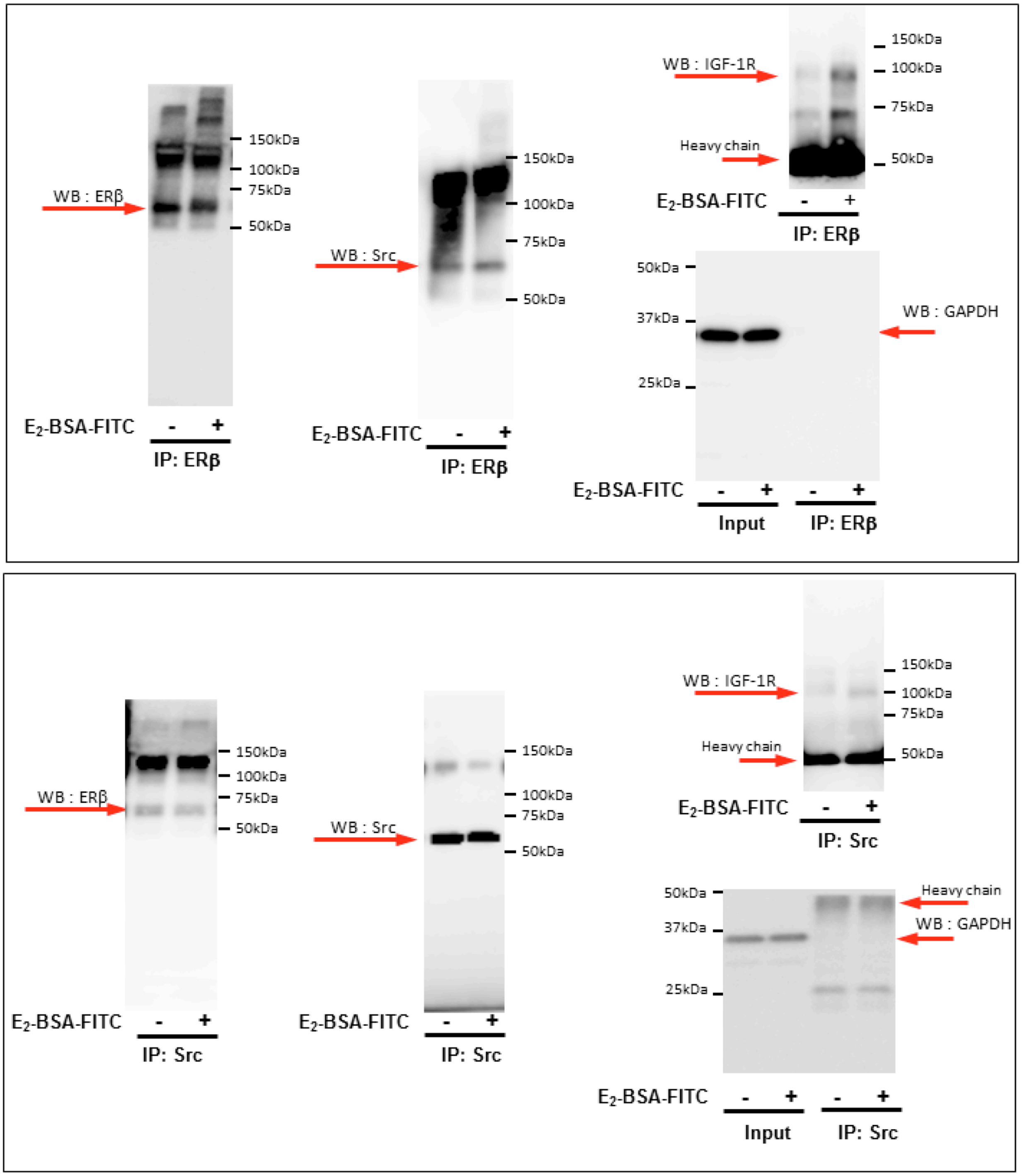

Supplementary Figure S6E: Whole membrane images for the Western blot and Co-IP experiments.

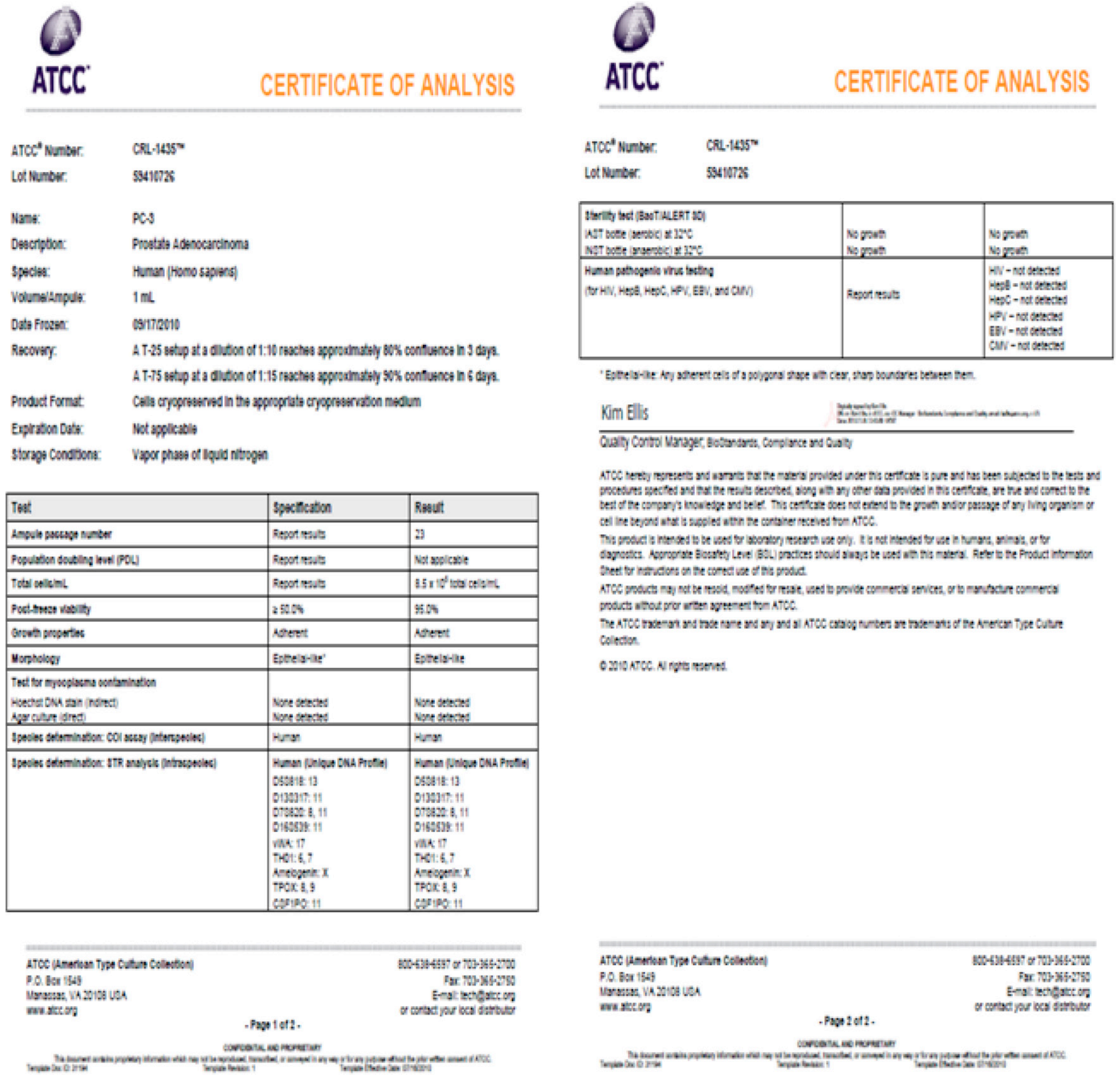

ATCC

CERTIFICATE OF ANALYSIS

ATCC® Number: CRL-1435™

Lot Number: 59410726

Name: PC-3

Description: Prostate Adenocarcinoma

Species: Human (Homo sapiens)

Volume/Ampule: 1 mL

Date Frozen: 09/17/2010

Recovery: A T-25 setup at a dilution of 1:10 reaches approximately 80% confluence in 3 days.
A T-75 setup at a dilution of 1:15 reaches approximately 90% confluence in 6 days.

Product Format: Cells cryopreserved in the appropriate cryopreservation medium

Expiration Date: Not applicable

Storage Conditions: Vapor phase of liquid nitrogen

Test	Specification	Result
Ampule passage number	Report results	23
Population doubling level (PDL)	Report results	Not applicable
Total cells/mL	Report results	8.5×10^{6} total cells/mL
Post-freeze viability	≥ 50.0%	95.0%
Growth properties	Adherent	Adherent
Morphology	Epithelial-like*	Epithelial-like
Test for mycoplasma contamination<br>Hoechst DNA stain (indirect)<br>Agar culture (direct)	 None detected<br>None detected	 None detected<br>None detected
Species determination: COI assay (interspecies)	Human	Human
Species determination: STR analysis (intraspecies)	Human (Unique DNA Profile)<br>D5S818: 13<br>D13S317: 11<br>D7S820: 8, 11<br>D16S539: 11<br>vWA: 17<br>TH01: 6, 7<br>Amelogenin: X<br>TPOX: 8, 9<br>CSF1PO: 11	Human (Unique DNA Profile)<br>D5S818: 13<br>D13S317: 11<br>D7S820: 8, 11<br>D16S539: 11<br>vWA: 17<br>TH01: 6, 7<br>Amelogenin: X<br>TPOX: 8, 9<br>CSF1PO: 11

ATCC (American Type Culture Collection)
P.O. Box 1549
Manassas, VA 20108 USA
www.atcc.org

800-638-6597 or 703-365-2700
Fax: 703-365-2750
E-mail: tech@atcc.org
or contact your local distributor

- Page 1 of 2 -

CONFIDENTIAL AND PROPRIETARY

This document contains proprietary information which may not be reproduced, transcribed, or conveyed in any way or for any purpose without the prior written consent of ATCC.

ATCC

CERTIFICATE OF ANALYSIS

ATCC® Number: CRL-1435™

Lot Number: 59410726

Sterility test (BacT/ALERT 3D)<br>iAST bottle (aerobic) at 32°C<br>iNST bottle (anaerobic) at 32°C	 No growth<br>No growth	 No growth<br>No growth
Human pathogenic virus testing<br>(for HIV, HepB, HepC, HPV, EBV, and CMV)	Report results	HIV – not detected<br>HepB – not detected<br>HepC – not detected<br>HPV – not detected<br>EBV – not detected<br>CMV – not detected

* Epithelial-like: Any adherent cells of a polygonal shape with clear, sharp boundaries between them.

Kim Ellis

Quality Control Manager, BioStandards, Compliance and Quality

ATCC hereby represents and warrants that the material provided under this certificate is pure and has been subjected to the tests and procedures specified and that the results described, along with any other data provided in this certificate, are true and correct to the best of the company's knowledge and belief. This certificate does not extend to the growth and/or passage of any living organism or cell line beyond what is supplied within the container received from ATCC.

This product is intended to be used for laboratory research use only. It is not intended for use in humans, animals, or for diagnostics. Appropriate Biosafety Level (BSL) practices should always be used with this material. Refer to the Product Information Sheet for instructions on the correct use of this product.

ATCC products may not be resold, modified for resale, used to provide commercial services, or to manufacture commercial products without prior written agreement from ATCC.

The ATCC trademark and trade name and any and all ATCC catalog numbers are trademarks of the American Type Culture Collection.

© 2010 ATCC. All rights reserved.

ATCC (American Type Culture Collection)
P.O. Box 1549
Manassas, VA 20108 USA
www.atcc.org

800-638-6597 or 703-365-2700
Fax: 703-365-2750
E-mail: tech@atcc.org
or contact your local distributor

- Page 2 of 2 -

CONFIDENTIAL AND PROPRIETARY

This document contains proprietary information which may not be reproduced, transcribed, or conveyed in any way or for any purpose without the prior written consent of ATCC.

Supplementary Figure S7: PC-3 cell line authentication from American Type Culture Collection (ATCC).
